# Cafeteria diet exposure, and not weight gain propensity, impacts gut microbiota of rats – a within laboratory meta-analysis

**DOI:** 10.1080/29933935.2026.2649442

**Published:** 2026-03-29

**Authors:** Shyam Prakaash Bhagavata Srinivasan, Michael D. Kendig, Kyoko Hasebe, Nadeem O. Kaakoush, Margaret J. Morris, Sarah-Jane Leigh

**Affiliations:** a Department of Pharmacology, School of Biomedical Sciences, UNSW, Sydney, NSW, Australia; b School of Life Sciences, UTS, Sydney, NSW, Australia; c Department of Pathology, School of Biomedical Sciences, UNSW, Sydney, NSW, Australia

**Keywords:** Meta-analysis, cafeteria diet, obesity, gut microbiota, obesity proneness, weight gain, sugar

## Abstract

Preclinical studies have implicated the microbiota in body weight control, but its translation to humans remains uncertain, partly owing to methodological variability in assessing the relationship between diet-induced obesity and microbiota composition. We performed an internal meta-analysis to determine whether the propensity for diet-induced obesity, defined by relative weight gain due to a high-fat, high-sugar “cafeteria” diet, is associated with changes in microbiota composition. We collated fecal microbiome data from 12 studies using our validated model of diet-induced obesity (208 male and 74 female Sprague–Dawley rats; 3.5–13 weeks of chow (control) or cafeteria diet) and determined whether the alpha diversity and composition of the gut microbiota differed between obese-prone and obese-resistant rats. We found consistent effects of cafeteria diet exposure on the microbiota, with marked changes in overall composition, and reduced microbial richness and evenness. Furthermore, specific obesity-associated microbial genera, such as *Bacteroides* and *Blautia*, were enriched by the cafeteria diet. Critically, alpha diversity measures and the gut microbiota composition did not differ between obese-prone and obese-resistant rats in either diet group. Our findings suggest that while the microbiota is substantially altered by cafeteria diet intake, these changes appear unrelated to individual susceptibility to weight gain, highlighting the role of additional host factors in modulating diet-induced obesity.

## 1. Introduction

The increase in obesity and metabolic syndrome has been linked to the consumption of a “western style” diet high in saturated fats and refined carbohydrates.^1^ It is well-established that diet is a major factor influencing the diversity and composition of the gut microbiota in both humans and rodents.^2^^,^^3^ Perturbations in the gut microbiota composition are associated with obesity and related metabolic diseases, including type 2 diabetes, non-alcoholic fatty liver disease, and cardio-metabolic diseases.^4^

Rodent models permit the study of the underlying mechanisms of obesity since experiments can be performed under strictly regulated conditions, and environmental factors can be well-controlled in a way that is not possible in long-term clinical studies. For many years, our laboratory has studied the behavioral, metabolic and neurobiological effects of a western style cafeteria diet,^5^^,^^6^ which consists of supplementing a standard rodent chow diet with a variety of palatable foods that are high in saturated fat and/or refined carbohydrates. The variety and palatability of the diet models the diversity of the modern diet and exposes rodents to a range of flavors, textures, nutrients, additives, sweeteners, and stabilizers consumed by most people. Cafeteria-style diets generate greater weight gain and more pronounced metabolic dysfunction than purified homogeneous high-fat diets (HFD),^7^ allowing rats to self-select their diets, although this comes at the expense of precise control over the intake of micronutrients and additives, which vary across foods.

Human and rodent obesity research has demonstrated substantial heterogeneity in diet-induced weight gain in subjects sharing similar genetic backgrounds and environments.^8^^,^^9^ These differences are often masked when data are presented as group means. However, some researchers have chosen to investigate individual differences in diet-induced weight gain; rodents with a predisposition to gain weight when challenged with an obesogenic diet can be defined as obese-prone, while those that exhibit reduced weight gain are defined as obese-resistant.^10^^,^^11^ Very few studies have compared the gut microbiota composition between obese-prone and obese-resistant subjects following prolonged obesogenic diet exposure. A recent study showed that male obese-prone C57BL/6J mice exhibited a distinct gut microbial and serum metabolic profiles relative to obese-resistant mice fed the same diet, and control diet mice after 8 weeks of HFD intake. Notably, *Parasutterella* abundance was elevated in obese-prone compared to obese-resistant and control diet-fed mice, which correlated positively with final body weight and serum metabolites involved in lipid and amino acid metabolism.^12^ In contrast, other studies have revealed no differences in gut microbial diversity or composition between obese-prone and resistant male Sprague-Dawley rats after 8 weeks of HFD,^13^ or in obese-prone and obese-resistant female C57BL/6J mice after 8 weeks of HFD.^14^ Discrepancies between these studies may be due to differences in gut microbial sequencing techniques, variation in gut microbiota composition and function along the gastrointestinal tract (i.e., caecum versus distal colon versus feces), the supplier and strain of animal used, diet type and duration, housing conditions and sex differences.

Here, we sought to explore this question in a combined analysis of studies from our lab showing altered gut microbiota diversity and composition following both brief and prolonged cafeteria diet exposure in female and male Sprague Dawley rats.^15-22^ To determine the relationship between diet-induced weight gain and gut microbiota composition in studies employing the same rat strain and obesogenic diet method, this internal meta-analysis combines data from rat experiments conducted in our laboratory since 2015. Although completed by different personnel, they were conducted in the same facility and conformed to the same procedures in terms of housing, cafeteria diet composition, and food intake measurement technique.^5^

While several meta-analyses of human and rodent studies have identified differences in gut microbiota diversity and composition with obesity or HFD exposure,^23-26^ to our knowledge, this is the first meta-analysis to investigate whether the propensity for diet-induced obesity is associated with changes in gut microbiota diversity and composition in rats. We examined whether changes in the gut microbiota composition in cafeteria and chow (control) diet-fed rats differed between obese-prone and obese-resistant rats, as determined by percentage body weight gain. We also examined the moderating influence of diet duration and sex on gut microbiota alpha and beta diversity parameters.

## 2. Methods

### 2.1. Study selection

All animal procedures in the included studies were approved by the Animal Care and Ethics Committee of UNSW Sydney in accordance with the Australian guidelines for the use and care of animals for scientific purposes 8th edition (National Health and Medical Research Council). The ethics approval numbers for all included studies are shown in Table 1. All animal experiments were carried out in compliance with the ARRIVE (Animals in Research: Reporting *In Vivo* Experiments) guidelines at the time.

**Table 1.** Characteristics of studies included in the meta-analysis.

Study ID	Sex	Diet duration (weeks)	Age at tissue collection (weeks)	Chow (*n*)	Cafeteria (*n*)	Year conducted	Reference	Ethics approval number
M 3.5	Male	3.5	16	10	8	2015	Beilharz et al. [15]	14/45B
M 3.5*	Male	3.5	16	12	12	2019	Kendig et al. [18]	17/65A
M 5	Male	5	18	12	12	2018	Kendig et al. [5]	17/65A
M 6	Male	6	14	12	12	2017	Leigh et al. [21]	16/67A &17/65A
M 7	Male	7	15	12	12	2016	Leigh et al. [20]	16/67A
F 7	Female	7	24	12	11	2017	Leigh et al. [19]	17/65A
M 8	Male	8	16	12	11	2017	Leigh et al. [21]	16/67A &17/65A
M 8*	Male	8	18	12	12	2018	Unpublished	16/67A &17/65A
M 11	Male	11	14	11	11	2019	Hasebe et al. [16]	19/74A
F 11	Female	11	14	11	11	2019	Hasebe et al. [16]	19/74A
M 13	Male	13	22	12	12	2021	Morris et al. [22]	20/113A
F 13	Female	13	17	14	15	2017	Kendig et al. [17]	16/67B

The column “Study ID” is formatted as follows for plotting purposes: Each study is labeled to show sex and diet duration in weeks; for example M 3.5 = male rats fed cafeteria diet for 3.5 weeks. Where two studies had the same sex and diet duration, an asterisk (*) is used to differentiate the studies. M = Male, F = Female.

This meta-analysis was restricted to datasets generated in our laboratory for both male and female Sprague-Dawley rats that met the following criteria: (1) used our validated cafeteria diet protocol^6^ supplemented with 10% sucrose, tap water and sourced the control diet from Gordon’s Stockfeeds, NSW, Australia; (2) used Sprague–Dawley rats sourced from the Animal Resource Centre, Perth, Australia; (3) group-housed rats (2–4 rats/cage) under similar conditions (temperature 18–22 °C, 12 h light/dark cycle); (4) involved continuous, uninterrupted access to cafeteria and control diets; (5) assessed retroperitoneal white adipose tissue (WAT) mass (g), and body weight (g) at the endpoint; (6) used similar DNA extraction protocols (PowerSoil or PowerFecal DNA isolation kit, Mo Bio Laboratories, Carlsbad, CA, USA); and (7) assessed microbiota composition using 16S rRNA Illumina amplicon sequencing (Ramaciotti Centre, UNSW Sydney). In cases where studies included groups to test other manipulations besides diet (e.g., exercise, drug/supplementation, or probiotic use), only samples from groups exposed to control or the cafeteria diet alone were included. Energy intake was measured once or twice a week across studies, assuming equal intake per rat in each cage. We included the unfasted insulin concentration (ng/ml) as an additional metabolic marker, which was also examined (6 of 12 studies).

### 2.2. Classifying susceptibility for weight gain

For each study, the rats in each diet group were classified into tertiles as obese-prone (top tertile), intermediate (middle tertile), or obese-resistant (bottom tertile) by percentage body weight gain from baseline (start of diet) to the endpoint (Supplementary Table 1). This method is widely used in the field to distinguish obese-prone and obese-resistant phenotypes in rodent models of diet-induced obesity.^9^^,^^10^^,^^12^^,^^14^^,^^27-29^ We further corrected for diet duration by dividing percentage body weight gain by the number of weeks on the cafeteria or control diet in each study. This allowed comparison across studies, as the duration of diet may impact both metabolic and adiposity endpoints, as well as the gut microbiota. Separation into tertiles generated the following groups: cafeteria diet obese-prone (Caf_Ob_, *n* = 35 males and 13 females), cafeteria diet intermediate (*n* = 33 males and 11 females), cafeteria diet obese-resistant (Caf_Res_, *n* = 35 males and 13 females), control diet obese-prone (C_Ob_, *n* = 35 males and 13 females), control diet intermediate (*n* = 35 males and 11 females), and control diet obese-resistant (C_Res,_ *n* = 35 males and 13 females) rats. There were more male than female rats in the included studies. We ensured that the ratio of males to females in each group was consistent.

To explore the impact of the cafeteria diet on adiposity measures and gut microbiota diversity and composition, we compared all rats fed the cafeteria diet against those fed the control diet. We determined the impacts of obesity proneness by comparing obese-prone, intermediate, and obese-resistant rats in each diet group, separately.

### 2.3. Statistical analyses

The sequencing data of each study were processed and analyzed using MOTHUR,^30^ described in detail by Hasebe et al.^16^ We followed the same protocol for all included studies, which included alignment with the SILVA database, checking chimaeras with UCHIME, and removal of singletons and classification against the latest RDP training set. These data were then rarefied to the lowest depth sample within each study and used for subsequent analyses (minimum reads/sample in each study are shown in Supplementary Table 10). We updated prokaryotic phylum names to reflect recent changes in validly published nomenclature.^31^ Older names such as *Firmicutes*, *Actinobacteria*, *Bacteroidetes*, and *Proteobacteria* were updated to *Bacillota*, *Actinomycetota*, *Bacteroidota*, and *Pseudomonadota*, respectively. All the statistical analyses explored sex-specific effects. We calculated alpha diversity metrics (species richness, evenness, Faith’s PD, and Shannon’s diversity index) for each study independently using PRIMER (Primer-7 Ltd., Plymouth, United Kingdom).^32^

GraphPad Prism 10 was used to generate and analyze the forest plots. The log_2_ fold change values were calculated against the geometric mean of the control diet or obese-resistant rats in each diet group. Statistical analysis was determined using the Wilcoxon *t-*test to determine significance and the 95% confidence interval (CI). The results are expressed as the mean ± 95% CI and were considered significant if *p* ≤ 0.05. Individual studies are referenced in the text using the sex of the subjects alongside the duration of the diet; for example, study F 11 used female rats that were fed a cafeteria diet for 11 weeks.

For specific bacterial taxonomic differences, data were analyzed at the genus level. The generated OTU relative abundance tables of each study were collapsed to the genus level by the addition of their respective relative abundances. Only genera that were present in all the experiments and exhibited global means greater than 0.01% were included in the analysis. Principal component analysis (PCA) was performed on centred log-ratio transformed (clr) values. Zeroes were replaced using the “const” approach described by Lubbe et al.^33^ Beta diversity was computed in terms of Aitchison distance, or the Euclidean distance between clr-transformed data, and assessed by Permutational Multivariate Analysis of Variance (PERMANOVA) and permutation multivariate analysis of dispersion (PERMDISP) was conducted with the function betadisper using the vegan and pairwise adonis packages. Statistical significance was defined as *p* ≤ 0.05.

The microbiota relative abundance data of all studies were combined at the genus level to identify differentially abundant genera between groups. We conducted MaAsLin3 (microbiome multivariable associations with linear models)^34^ using R (v4.5.2;^35^ MaAsLin3 analyses enables the identification of differentially abundant genera while accounting for covariates and data compositionality.^34^ We used default settings to perform the analyses: the relative abundance data underwent total-sum scaling (TSS) and log transformation; only microbial genera detected in at least 10% of the samples were tested; no minimum abundance or prevalence was specified; and the significance threshold was set as *p* ≤ 0.05 and *q* ≤ 0.25 (corrected *p*-value by Benjamini-Hochberg procedure). Coefficients were estimated using both abundance and prevalence models. The individual *q*-value reflects the significance of associations with either differential abundance or prevalence of a given microbial genus, while the joint *q*-value assesses the significance of associations considering both abundance and prevalence. Features assigned as “error” were excluded, and only associations with a joint *q* ≤ 0.10 were considered significant. To determine the impact of the cafeteria diet in the model, we included diet as a fixed effect and specified sequencing depth (minimum reads/sample), study, cage, sex, sequencing platform, duration of diet (weeks), and age at cull (weeks) as covariates. To determine the impacts of obesity proneness, we conducted the analyses in each diet group separately. We included obesity proneness (obese-prone, intermediate, and obese-resistant) as a fixed effect and specified sequencing depth (minimum reads/sample), study, cage, sex, sequencing platform, duration of diet (weeks), and age at cull (weeks) as covariates.

## 3. Results

### 3.1. Study characteristics

A total of 12 studies with 282 Sprague–Dawley rats (208 males, 74 females) were included (Table 1). This included one unpublished study. One study sequenced a different hypervariable region of the 16S rRNA gene (V1–V3 region, 27F-519R primer pair)^15^ while all other studies sequenced the same region (V4 region, 515F-806R primer pair). Six of the 12 studies collected data on insulin concentration at the endpoint. The studies also varied by diet duration (3.5–13 weeks) and age at tissue collection (14–24 weeks). The relevant study details are shown in Table 1. As shown in the ternary plot (Supplementary Figure 1), the macronutrient composition (by % kJ) of the control diet was consistent across all studies (65% carbohydrate, 22%–23% protein and 12%–13% fat, kJ). As expected, the cafeteria diet contained increased dietary fat, but this varied between studies (29%–40% kJ). The protein content in the cafeteria diet was lower (8%–13% kJ), and the carbohydrate content was mostly consistent across studies (55%–60.8% kJ). The average 24-h energy intake (kJ/rat) was higher in the cafeteria diet compared to control diet groups (Supplementary Figure 2; Supplementary Table 2).

### 3.2. Influence of diet and obesity proneness on adiposity measures

**Figure 1.**
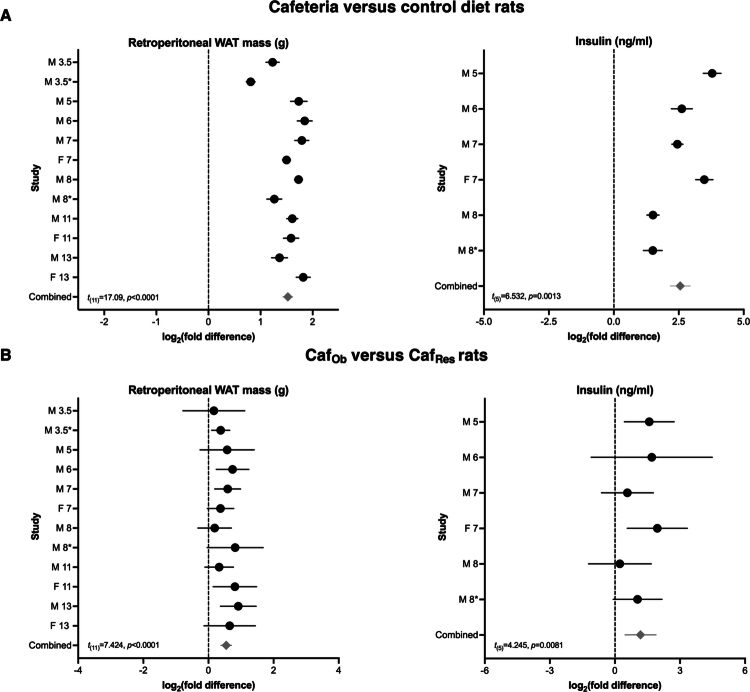
Measures of adiposity (retroperitoneal WAT mass and insulin concentration) are influenced by diet and obesity-proneness. (A) Forest plots comparing cafeteria and control diet-fed rats. The values were scaled to the geometric mean of the control diet samples on a per-study basis. Cafeteria diet: *n* = 103 males (M), 37 females (F), and control diet: *n* = 105 males, 37 females. (B) Forest plots comparing cafeteria diet obese-prone (Caf_Ob_) and cafeteria diet obese-resistant (Caf_Res_) rats. The values were scaled to the geometric mean of obese-resistant rats on a per-study basis. The rats were classified into tertiles as obese-prone, intermediate, and obese-resistant based on percentage weight gain (corrected for weeks of diet) from the start of diet to endpoint (cull). Caf_Ob_: *n* = 35 males, 13 females; Caf_Res_: *n* = 35 males, 13 females. The data are displayed as the mean  ±  95% CI and were analyzed via the Wilcoxon *t*-test (*p*-values are indicated on each plot). The mean effect size (log_2_(fold difference)) is shown as black circles, and the lines indicate the confidence interval. The overall effect size is indicated by the gray diamond. Each study is labeled as specified in Table 1 to show sex and diet duration in weeks; for example, M 3.5 = male rats fed a cafeteria diet for 3.5 weeks. *Indicates a second study of the same sex and diet duration. WAT = white adipose tissue, ng = nanograms, ml = milliliters, g = grams.

The endpoint average retroperitoneal WAT mass and unfasted insulin concentrations between the diet groups are shown in Supplementary Figure 3 and Supplementary Table 3. The retroperitoneal WAT mass was consistently increased by the cafeteria diet across all the studies (95% CI: 1.33, 1.72; *t*_(11)_ = 17.09, *p* < 0.0001) (Figure 1A; Supplementary Table 3). In the six studies where the insulin concentration at the endpoint was available, a significant increase was observed with the cafeteria diet (95% CI: 1.55, 3.57, *t*_(5)_ = 6.532, *p* = 0.0013) relative to the control (Figure 1A; Supplementary Table 3).

To investigate the impact of obesity-proneness, the rats from each diet group were classified as obese-prone, intermediate, or obese-resistant based on percentage body weight gain. Following classification, percentage weight gain was significantly greater in Caf_Ob_ versus C_Res_ rats (95% CI: 0.44, 0.64, *t*_(11)_ = 11.40, *p* < 0.0001; Supplementary Figure 4), and in C_Ob_ versus C_Res_ rats (95% CI: 0.42–0.87, *t*_(11)_ = 6.293, *p* < 0.0001; Supplementary Figure 4). The average percentage weight gain of each group is shown in Supplementary Figure 3. Both retroperitoneal WAT mass and insulin concentration at the endpoint were significantly elevated in Caf_Ob_ rats compared with Caf_Res_ rats (Figure 1B; Supplementary Table 5) and in C_Ob_ rats compared with C_Res_ rats (Supplementary Figure 5 A; Supplementary Table 7). Our data showed that retroperitoneal WAT mass was relatively higher in Caf_Ob_ versus Caf_Res_ rats (combined log_2_ fold difference = 0.54) than in C_Ob_ rats compared with C_Res_ rats (combined log_2_ fold difference = 0.36).

### 3.3. Cafeteria diet alters gut microbial alpha diversity

**Figure 2.**
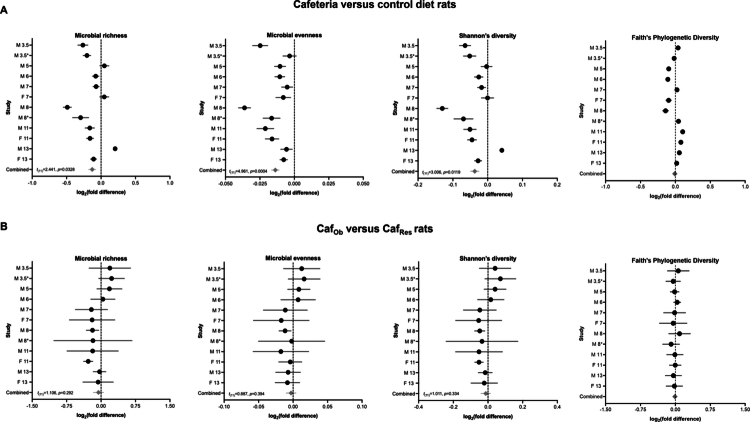
Influence of diet and obesity-proneness on alpha diversity metrics: microbial richness, microbial evenness, Shannon’s diversity index, and Faith’s phylogenetic diversity. (A) Forest plots comparing cafeteria and control diet-fed rats. The values were scaled to the geometric mean of the control diet samples on a per-study basis. Cafeteria diet: *n* = 103 males (M), 37 females (F), and control diet: *n* = 105 males, 37 females. (B) Forest plots comparing cafeteria diet obese-prone (Caf_Ob_) and cafeteria diet obese-resistant (Caf_Res_) rats. The values were scaled to the geometric mean of obese-resistant rats on a per-study basis. The rats were classified into tertiles as obese-prone, intermediate, and obese-resistant based on percentage weight gain (corrected for weeks of diet) from the start of diet to endpoint (cull). Caf_Ob_: *n* = 35 males, 13 females; Caf_Res_: *n* = 35 males, 13 females. The data are displayed as the mean ± 95% CI and were analyzed via the Wilcoxon *t*-test (*p*-values are indicated on each plot). The mean effect size is shown as black circles, and the lines indicate the confidence interval. The overall effect size is indicated by the gray diamond. Each study is labeled as specified in Table 1 to show sex and diet duration in weeks; for example, M 3.5 = male rats fed a cafeteria diet for 3.5 weeks. *Indicates a second study of the same sex and diet duration.

Our combined analyses identified significant differences between rats fed cafeteria diet versus control diet on in all alpha diversity metrics except Faith’s phylogenetic diversity, as shown in Figure 2A. The gut microbial richness (95% CI: −0.24, −0.01, *t*_(11)_ = 2.441, *p* = 0.0328) was decreased in 9 out of 12 studies, and Shannon’s diversity index (95% CI: −0.06, −0.01, *t*_(11)_ = 3.006, *p* = 0.0119) was decreased in 10 out of 12 studies, and the gut microbial evenness (95% CI: −0.02, −0.01, *t*_(11)_ = 4.961, *p* = 0.0004) was decreased in all studies with the cafeteria diet relative to the control diet (Figure 2A; Supplementary Table 4). We then explored the impacts of obesity-proneness and found no significant differences in alpha diversity between obese-prone and obese-resistant rats in each diet group when analyzed separately (Caf_Ob_ vs. Caf_Res_, Figure 2B and Supplementary Table 6) (C_Ob_ vs. C_Res_, Supplementary Figure 5B and Supplementary Table 8).

### 3.4. Gut microbiota composition is influenced by diet but not by propensity for weight gain

**Figure 3.**
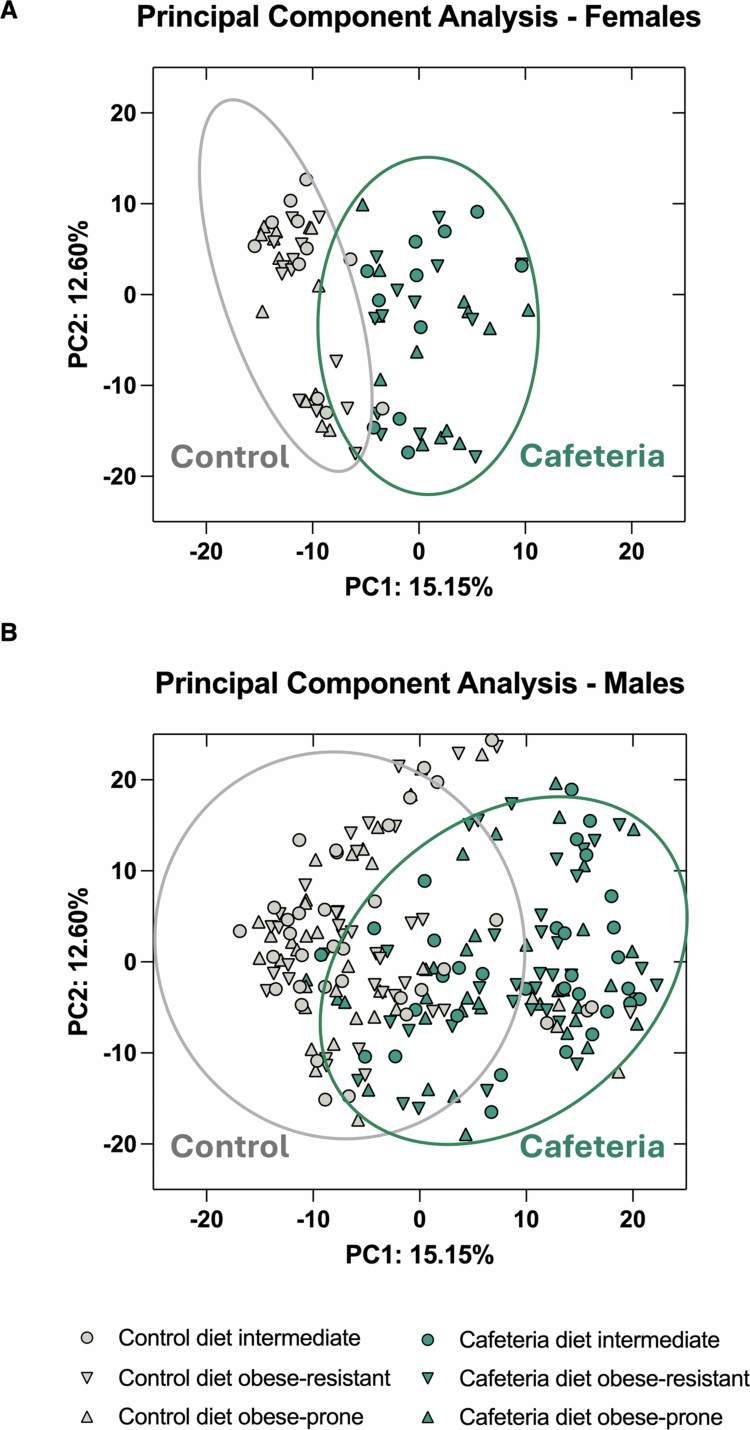
Principal component analysis (PCA) plots illustrating the impacts of diet and obesity-proneness on the overall gut microbiota composition at the genus level separated by sex. (A) Females and (B) males. Each point represents an individual rat; the ellipse represents the 95% confidence interval. Cafeteria diet obese-prone (green triangle; *n* = 35 males and 13 females), cafeteria diet intermediate (green circle; *n* = 33 males and 11 females), cafeteria diet obese-resistant (inverted green triangle; Caf_Res_, *n* = 35 males and 13 females), control diet obese-prone (gray triangle; C_Ob_, *n* = 35 males and 13 females), control diet intermediate (gray circle; *n* = 35 males and 11 females), and control diet obese-resistant (inverted gray triangle; C_Res,_ *n* = 35 males and 13 females) rats. The rats were classified into tertiles as obese-prone, intermediate, and obese-resistant based on percentage weight gain (corrected for weeks of diet) from the start of diet to endpoint (cull).

We next explored whether the beta diversity of the gut microbiota was altered by the cafeteria diet and obesity-proneness. Visualization by PCA plot of all 282 rats demonstrated a clear separation between cafeteria and control diet-fed rats (Figures 3A, B; Supplementary Figure 6). We then assessed differences in microbiota composition using a three-way PERMANOVA (permutations 999) and found that diet, sex, and diet duration exhibited a significant three-way interaction (*F*_(1,274)_ = 2.142, *p* = 0.008). Pairwise comparisons showed that all groups were significantly different from one another (all *p* < 0.005), except cafeteria diet males versus control diet males in study M 3.5^15^ (*F*_(1,40)_ = 1.608, *p* = 0.109), control diet females in study F 11^16^ versus control diet males in study M 11^16^ (*F*_(1,20)_ = 1.254, *p* = 0.163), and cafeteria diet females in study F 11^16^ versus cafeteria diet males in study M 11^16^ (*F*_(1,20)_ = 0.844, *p* = 0.657). The microbial communities of control diet-fed rats were more dispersed than those of cafeteria diet-fed rats (PERMDISP: *F*_(19,262)_ = 77.465, *p* < 0.001).

We assessed whether the overall gut microbiota composition was influenced by obesity proneness by pairwise PERMANOVA (C_Ob_ vs. control diet intermediate vs. C_Res_ vs. Caf_Ob_ vs. cafeteria diet intermediate vs. Caf_Res_), we detected significant differences between rats fed control diet and each cafeteria diet-fed group (all *p* = 0.001; Supplementary Table 9); however, no significant differences between obese-prone and obese-resistant rats were observed in either the C_Ob_ versus C_Res_ (*F*_(1, 94)_ = 0.459, *p* = 0.999), or Caf_Ob_ versus Caf_Res_ rats (*F*_(1, 94)_ = 0.720, *p* = 0.846) (Figures 3A, B).

### 3.5. Specific microbial taxa are influenced by cafeteria diet

**Figure 4.**
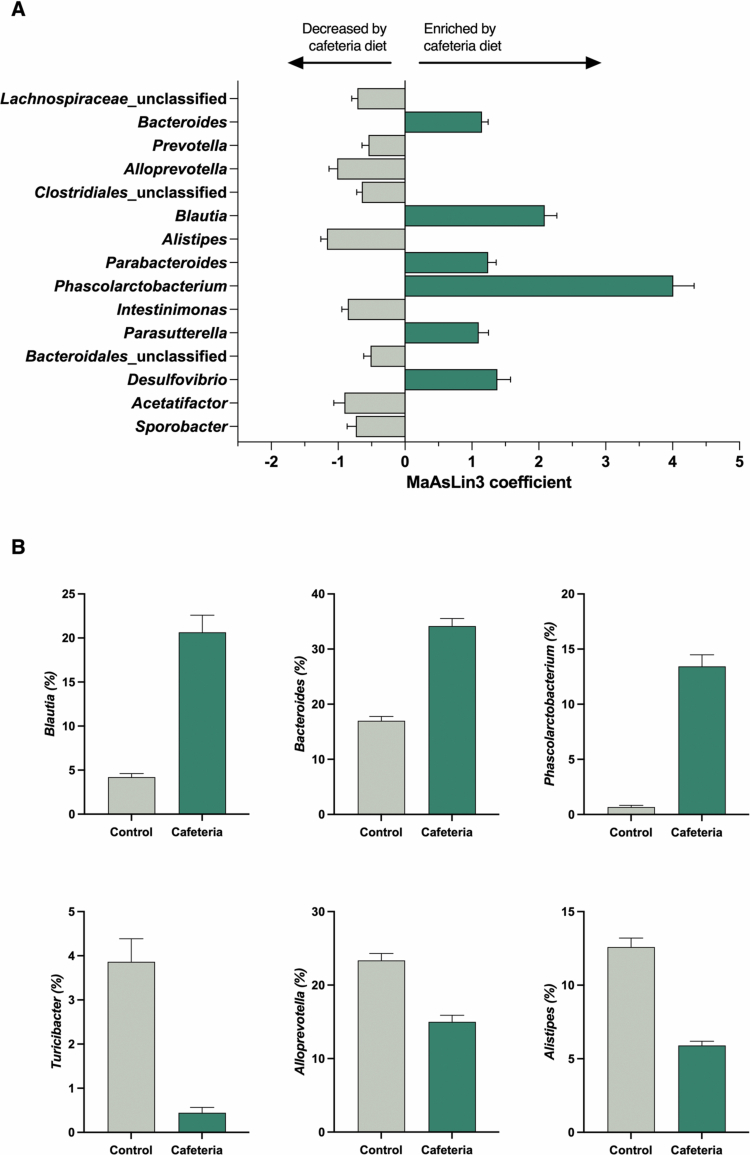
The cafeteria diet altered the abundance of individual gut microbial genera. (A) Relative abundance of the top 15 microbial genera identified to be significantly different between cafeteria and control diet-fed rats by MaAsLin3 analyses (*q* ≤ 0.10). The model was adjusted for sequencing depth (reads/sample), study, cage, sex, sequencing platform, duration of diet (weeks), and age at cull (weeks). The genera enriched by the cafeteria diet are shown in green with bars to the right whilst those enriched by the cafeteria diet are shown in gray with bars to the left. The length of the bars corresponds to the MaAsLin3 coefficient, and the error bars indicate the standard error of the model coefficient. (B) The percentage relative abundance of six microbial genera identified to be significantly different between cafeteria and control diet-fed rats by MaAsLin3 analyses. The data are presented as the mean ± SEM. Cafeteria diet: *n* = 103 males (M), 37 females (F), and control diet: *n* = 105 males, 37 females. MaAsLin3 = Microbiome multivariable associations with linear models.

By combining the gut microbial composition data of all the studies at the genus level, we explored whether specific microbial taxa differed between the cafeteria and control diet groups by MaAsLin3 analyses. We included sequencing depth (reads/sample), study, cage, sequencing platform, sex, duration of diet (weeks), and age at cull (weeks) as covariates. Our analyses identified 66 genera that were differentially abundant between cafeteria and control diet-fed rats (*q* ≤ 0.10; Figures 4A, B; Supplementary Table 11), with 32 genera that increased and 34 decreased in abundance in response to cafeteria diet intake. Among those identified, the genera *Bacteroides* (coefficient = 1.15, *q* = 8.33 × 10^−11^), *Blautia* (coefficient = 2.09, *q* = 1.18 × 10^−13^), *Phascolarctobacterium* (coefficient = 4.01, *q* = 5.85 × 10^−16^), and *Parabacteroides* (coefficient = 1.24, *q* = 5.49 × 10^−10^) were enriched, while *Turicibacter* (coefficient = −1.81, *q* = 5.82 × 10^−11^)*, Intestinimonas* (coefficient = −0.86, *q* = 3.96 × 10^−6^), *Alloprevotella* (coefficient = −1.01, *q* = 2.07 × 10^−6^), and *Alistipes* (coefficient = −1.17, *q* = 1.13 × 10^−9^) were decreased by cafeteria diet (Figure 4A, B).

To explore the impacts of obesity proneness, we compared obese-prone, intermediate, and obese-resistant rats in each diet group separately. We included sequencing depth (reads/sample), study, cage, sex, sequencing platform, duration of diet (weeks), and age at cull (weeks) as covariates. These analyses revealed no significant differences (all *q* > 0.10). Interestingly, we identified a non-significant drop (at *q* < 0.25) in the relative abundance of the genera *Collinsella* (coefficient = −0.62, *q* = 0.172) and *Escherichia/Shigella* (coefficient = −0.38, *q* = 0.164) in Caf_Res_ rats relative to cafeteria intermediate rats (Supplementary Figure 7). As shown in Supplementary Figure 7, the relative abundance of *Collinsella* was also reduced to a similar extent in Caf_Ob_ rats relative to cafeteria intermediate rats (coefficient = −0.75, *q* = 0.245).

## 4. Discussion

This internal meta-analysis of 282 rats fed obesogenic or control diets shows strong effects of diet on gut microbiota diversity and composition, with no effects of propensity for weight gain. Cafeteria diet intake consistently and robustly reduced gut microbial alpha diversity and led to significant overall changes in the composition of the global gut microbiota. We identified significant effects of cafeteria diet exposure on several microbial genera, including *Bacteroides* and *Blautia*, which have been consistently associated with obesity/cafeteria diet intake previously.^36^^,^^37^ Notably, classifying rats as obese-prone or obese-resistant based on relative weight gain did not reveal significant differences in gut microbial alpha diversity or composition in both diet groups, despite increased retroperitoneal WAT mass and plasma insulin in obese-prone versus obese-resistant rats. Thus, diet appears to be the major driver of gut microbiota differences.

Our findings are in line with de La Serre et al.^13^, who found that male Sprague–Dawley rats presented reduced gut microbial alpha diversity and changes in composition after 8 weeks of HFD intake, but no differences in gut microbiota diversity or composition between obese-prone and obese-resistant subgroups^13^ as well as work using female C57BL/6 mice given 8 weeks of HFD access.^14^ However, several studies have found differences in microbiota diversity and composition between HFD-fed obese-prone and resistant rodents.^12^^,^^38^^,^^39^ Here, we assessed the effects of obesity-proneness and resistance across multiple studies to maximize power: obese-proneness approaches are often not adopted owing to large animal requirements and wastage (since one-third of rats are discarded from the analysis), or conducted with rats bred for this phenotype,^10^ which necessitates intergenerational HFD exposure, which may independently alter obesity susceptibility.^40^^,^^41^ By restricting our meta-analysis in-house, we were able to control for confounding factors which may explain the discrepancies between studies on this question. All 12 cohorts used the same validated rodent model of cafeteria diet-induced obesity,^6^ the same rodent and food vendors, food intake measurement technique, animal strain, housing conditions, sample collection, and DNA extraction technique.

We classified rats into tertiles based on percentage weight gain in response to the cafeteria diet, a widely used approach in rodent models of diet-induced obesity to identify obese-prone and obese-resistant phenotypes at the extremes of the weight gain distribution.^9^^,^^10^^,^^12^^,^^14^^,^^27-29^ Though this approach may limit insights into more subtle, continuous relationships with weight gain, it allowed for a clearer interpretation of group-level differences and alignment with prior literature.

Whilst the exclusive use of Sprague–Dawley rats eliminates strain differences as a potential confounding factor, it reduces the generalizability of our findings to other commonly used rodent strains, such as Wistar rats or mouse models like C57BL/6, which may differ in their susceptibility to diet-induced obesity.^42^^,^^43^ Outbred Sprague–Dawley rats are commonly used to study obesity proneness in diet-induced obesity due to their genetic and epigenetic variability, which mirrors human susceptibility to obesity.^9^^,^^10^ Another important consideration is the use of the same cafeteria diet protocol across all included studies. The cafeteria diet is well-established in our laboratory, closely mimicking the variety and palatability of the human western diet.^6^ The diet is particularly relevant for microbiota work, as it includes common dietary additives that can alter microbiota composition and function that are found in obesogenic human foods (through the incorporation of human foods)^6^ and induces greater alterations in microbiota composition relative to other obesogenic diet exposures in rats.^44^ However, it does differ from the purified HFD and western diet models commonly used in other studies, which may limit the direct comparability of our findings. One key limitation of the cafeteria diet is the range of macronutrient profiles observed, as individual rats select the foods that they consume. In this meta-analysis, we observed ranges of 8%–13% protein, 50%–60.8% carbohydrate and 29%–40% fat intake. These differences in macronutrient profiles likely impact microbiota composition.^18^^,^^45-47^ A clear future direction will be to directly compare the impacts of the cafeteria diet and purified HFD models on the gut microbiota, an analysis that was beyond the scope of the present meta-analysis.

Our goal was to identify whether changes in specific microbial taxa could explain proneness or resistance to diet-induced weight gain. We found no evidence for this, with no taxa differing statistically between obese-prone and obese-resistant rats. The lack of identifiable links between specific microbiota and body weight changes is in line with human studies that show inconsistent effects of microbiota interventions such as prebiotics, probiotics, and synbiotics on body weight gain/loss in obesity.^48-51^ Further studies are required to identify the mechanisms driving the individual differences in weight gain observed in response to an obesogenic diet. Interestingly, Tran et al.^14^ found taxonomic differences in the faecal metaproteome of HFD-fed obese-prone and resistant female mice despite finding no differences in the gut microbiota composition. This suggests that there may be functional differences in the gut microbiota of obese-prone versus obese-resistant rats in this study that are not accessible via targeted sequencing. Other studies in rodents and humans have suggested a possible relationship between a HFD and gut-derived metabolites such as faecal bile acids,^38^ branched fatty esters of hydroxy fatty acids,^39^ polyphenol metabolites such as *p*-hydroxyphenylacetic acid,^52^ and short-chain fatty acids such as propionate^53^ on susceptibility for weight gain. Furthermore, most research into obesity and the gut microbiota has focused on the bacteriome, but emerging data indicate that the gut virome^54^ and mycobiome^55^ may also be involved in the host response to a poor diet. As our understanding of the microbiota develops, it is likely that other factors contribute to host‒microbiome interactions in the context of obesity.

Consistent with the existing literature, our analyses found that consumption of the cafeteria-style western diet reduced gut microbial alpha diversity and altered the composition of the gut microbiota. Similar results were also shown with a HFD in a recent meta-analysis of 25 rodent studies.^23^ Although the cohort was male-skewed (208 males versus 74 females), we included female rodents to improve the representation of both sexes and accounted for sex as a covariate in all statistical analyses to mitigate potential confounding. Importantly, our data showed that cafeteria diet consumption produced consistent effects on the gut microbiota across both sexes. This is a critical finding given that limited preclinical research has examined the impact of poor diet on female microbiota composition and function. However, as this study was not specifically designed to assess sex-based differences in the gut microbiota or metabolic responses, future work is needed to explore these interactions in more detail. We found an increased abundance of the genus *Bacteroides* following cafeteria diet, which agrees with several human studies after consumption of diets high in saturated fat diet.^37^^,^^56-58^ Similarly, our finding of enriched *Blautia* is in line with other studies of cafeteria diet/HFD intake.^36^^,^^59^^,^^60^ At the genus level, enriched *Blautia* has been associated with elevated levels of obesity-related plasma metabolites,^61^ and development of type 2 diabetes.^62^ However, the interpretation of this finding requires species-level nuance.^63^ For instance, certain species of *Blautia* have been shown to play a beneficial role in preventing intestinal inflammation by upregulating intestinal regulatory T cells and producing short-chain fatty acids such as propionate and acetate.^64^ Notably, *Blautia hansenii* was associated with reduced adiposity in a recent longitudinal study of 767 Japanese individuals aged 20–76 y,^65^ suggesting a potentially protective role. These findings highlight the importance of taxonomic resolution, as genus-level analyses may obscure species-specific effects that differentially influence host metabolic outcomes. It is important for future studies to investigate whether diet-induced microbiota changes act as a biomarker or underlie other effects of poor diet on host physiology and health.

Given that this meta-analysis was conducted in-house, further work is needed to confirm our findings in larger cohorts. We combined sequencing data at the genus level to characterize the composition of the gut microbiota across studies using different 16S sequencing primers. Additional changes in the gut microbiota composition may have been detected at the species level, or through shotgun metagenomics sequencing, which would have also provided functional resolution. For instance, Wei et al.^38^ identified elevated levels of *Clostridium scindens* and *Clostridium hylemonae* in obese-prone versus resistant male C57BL/6 mice after 82 weeks of HFD. In agreement with our findings, they found no differences in alpha diversity or changes in composition at higher taxonomic levels between obese-prone and obese-resistant mice. A clear future direction will be to compare our results with external datasets from other laboratories and incorporate metagenomic analyses. A further consideration is the variability in rarefaction depth across studies, which may have influenced alpha diversity estimates and the detection of low-abundance taxa.^66^ Although analyses were conducted at the genus level and adjusted for study and sequencing depth to mitigate these effects, future work using consistent 16S sequencing primers, sequencing workflows, and analysis approaches may further improve cross-study comparisons.

Although we corrected for diet duration, the included studies had a wide range of diet durations from 3.5 to 13 weeks. Interestingly, our data showed a possible effect of diet duration on gut microbial alpha diversity in Caf_Ob_ rats relative to Caf_Res_ rats, with a trend toward increased diversity in studies involving less than 6 weeks of a cafeteria diet and reduced diversity in those involving more than 7 weeks of a cafeteria diet (Figure 2B). More studies are needed to examine the time-course effects of poor diet consumption on the gut microbiota. Existing evidence suggests resilience of the gut microbiota to short-term dietary interventions in humans and rodents,^2^^,^^67^^,^^68^ but the extent of diet exposure required to induce irreversible and deleterious effects on gut microbiota composition and function is unknown. An important factor that we were insufficiently powered to consider was early-life exposure to a poor diet and its effects on microbial species diversity and microbiota composition. In this analysis, only two studies in which diet was initiated prior to adulthood were included,^16^^,^^17^ since early childhood and adolescence are important developmental windows for the gut microbiota,^69^ and poor diet during these stages is likely to exert exaggerated or disparate effects. Furthermore, these rats gained substantially more weight during dietary exposure, since they were actively growing as well as gaining adipose tissue. Another critical, understudied area is how poor diet interacts with diurnal rhythmicity to shape microbiota composition: since the gut microbiota exhibits significant shifts in alpha and beta diversity across the day^70^^,^^71^^,^^72^ and can alter how the microbiota shapes host physiology,^71^^,^^73^ it is likely that these changes will influence the magnitude of diet-induced changes, particularly in the context of studies with shorter diet durations, or timed exposure. In the studies represented, microbiota samples were collected at a similar time during the light phase (Zeitgeiber time 2–8), mitigating some of this variance. However, more systematic studies into how microbial diurnal rhythmicity is disrupted by cafeteria diets are warranted.

## 5. Conclusions

In conclusion, this meta-analysis of 12 studies showed that the diversity and composition of the gut microbiota of Sprague–Dawley rats are substantially altered by cafeteria diet intake but are unrelated to their susceptibility for weight gain. We found that the abundance of specific genera, such as *Bacteroides* and *Blautia,* which have been previously associated with poor diet/obesity, were consistently altered by cafeteria diet intake; however, more mechanistic studies are needed to establish a causal link. By restricting our meta-analysis to studies collected in our laboratory, we were able to control for the technical variations commonly encountered when comparing microbiome studies. Such data allows us to facilitate more robust biological conclusions that can be further investigated in larger cohorts.

## Supplementary Material

Figure S2.jpg Figure S2.jpg

Supplementary Table 1.docx Supplementary Table 1.docx

Figure S4.jpg Figure S4.jpg

Figure S3.jpg Figure S3.jpg

Supplementary Table 2.docx Supplementary Table 2.docx

Supplementary Table 5.docx Supplementary Table 5.docx

Supplementary Table 11.docx Supplementary Table 11.docx

Figure S7.jpg Figure S7.jpg

Supplementary Table 8.docx Supplementary Table 8.docx

Supplementary Table 4.docx Supplementary Table 4.docx

Supplementary Table 7.docx Supplementary Table 7.docx

Figure S6.jpg Figure S6.jpg

Supplementary Table 6.docx Supplementary Table 6.docx

Figure S5.jpg Figure S5.jpg

Supplementary Table 10.docx Supplementary Table 10.docx

Figure S1.jpg Figure S1.jpg

Supplementary Table 3.docx Supplementary Table 3.docx

Supplementary Table 9.docx Supplementary Table 9.docx

## Data Availability

Sequence data from published studies included in this meta-analysis are available in the European Nucleotide Archive under accession numbers: PRJEB32323, PRJEB34488, PRJEB36541, and PRJEB46141. Sequence data from unpublished studies will be made available by the corresponding author upon reasonable request. All other data, including sample metadata and statistical analysis outputs, will be made available upon request from the corresponding author upon reasonable request. The timeframe for providing these materials will be determined based on the nature and scope of the request, ensuring timely and appropriate access.
