# Supplementary figures and images for: Cafeteria diet exposure, and not weight gain propensity, impacts gut microbiota of rats – a within laboratory meta-analysis
Source: Gut Microbes Rep. 2026 Mar 29;3(1):2649442. doi: 10.1080/29933935.2026.2649442 (PMC13037442; doi:10.1080/29933935.2026.2649442)

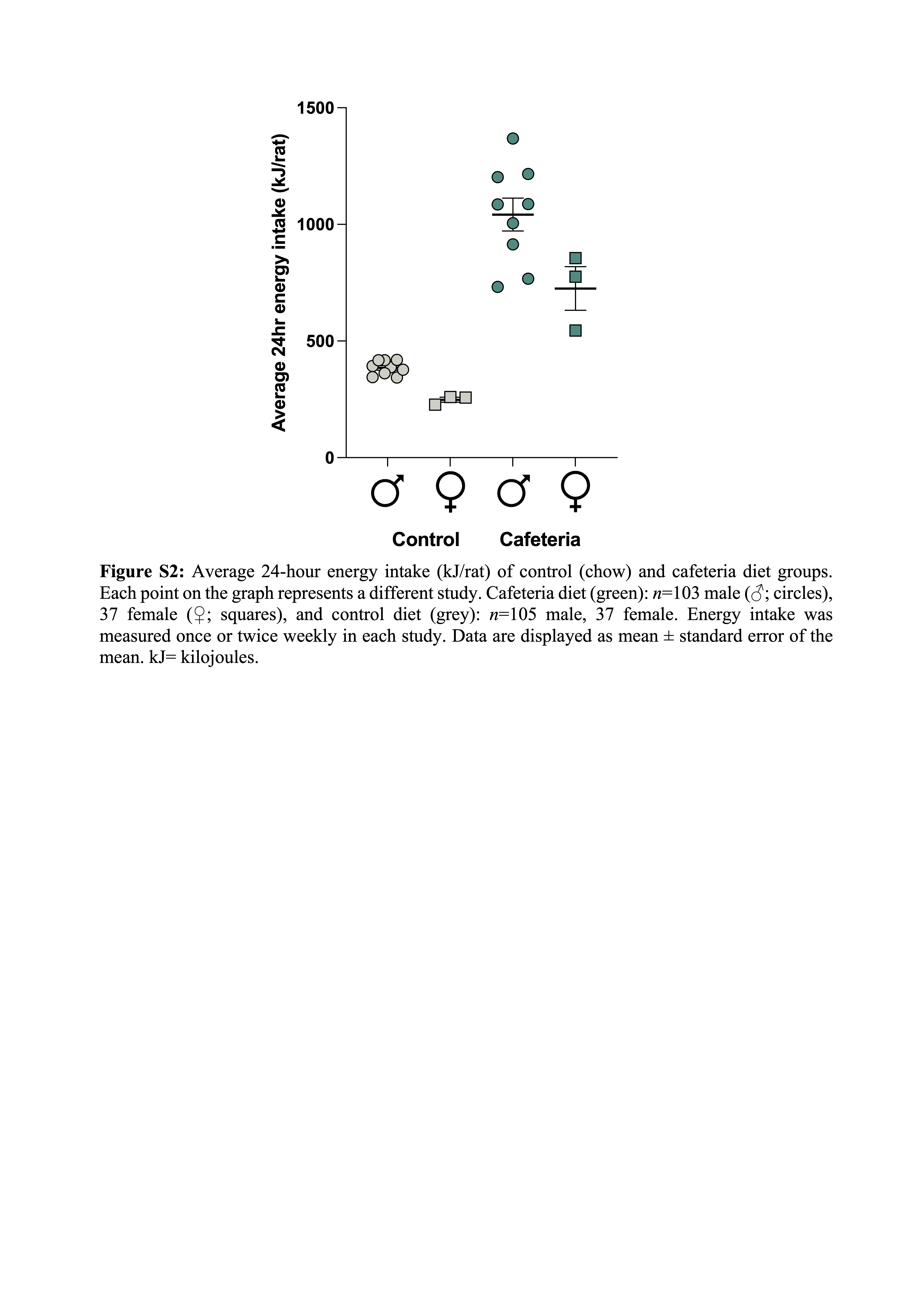

Supplement: Figure S2.jpg [file KGMR_A_2649442_SM2622.jpg]

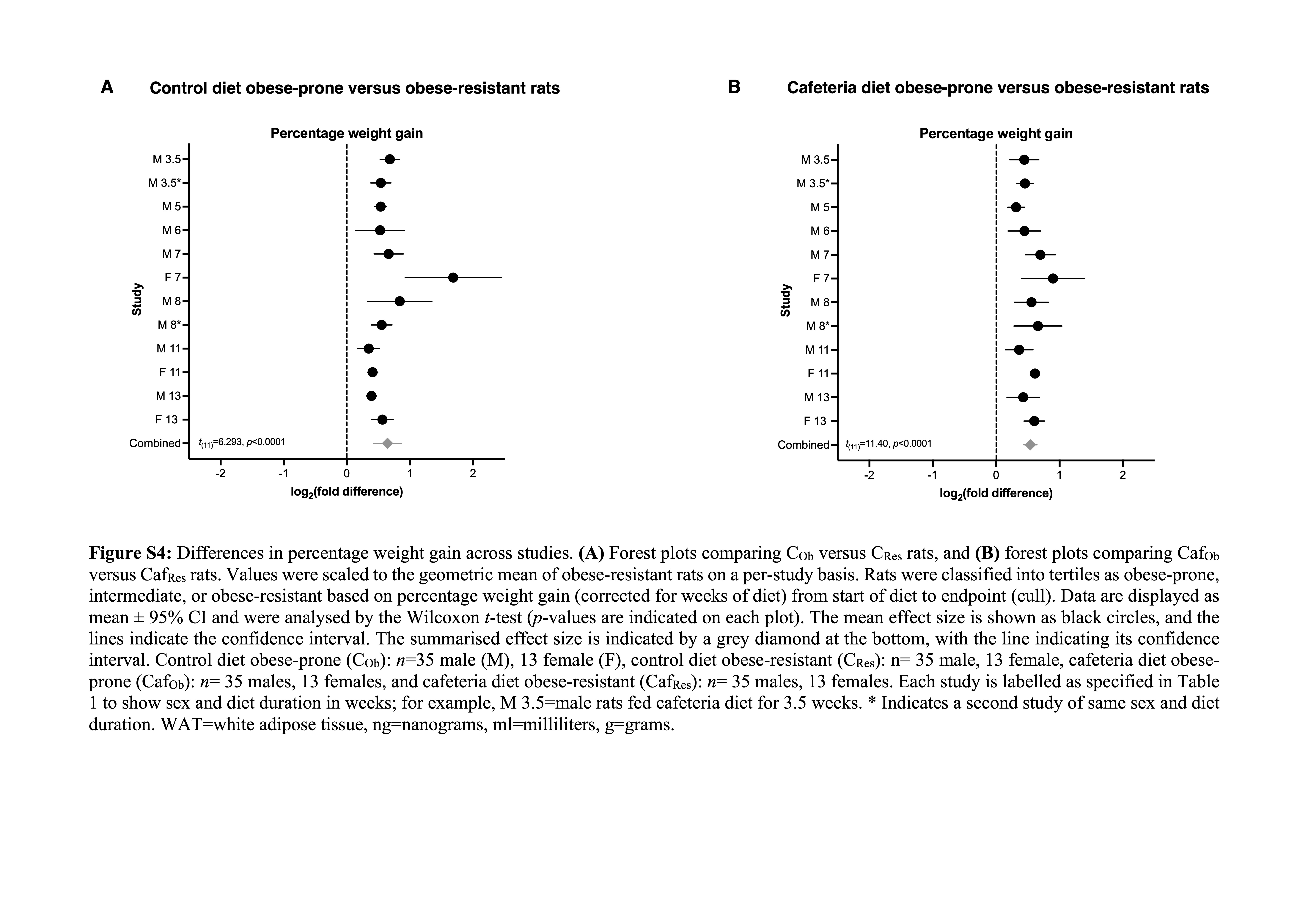

Supplement: Figure S4.jpg [file KGMR_A_2649442_SM2605.jpg]

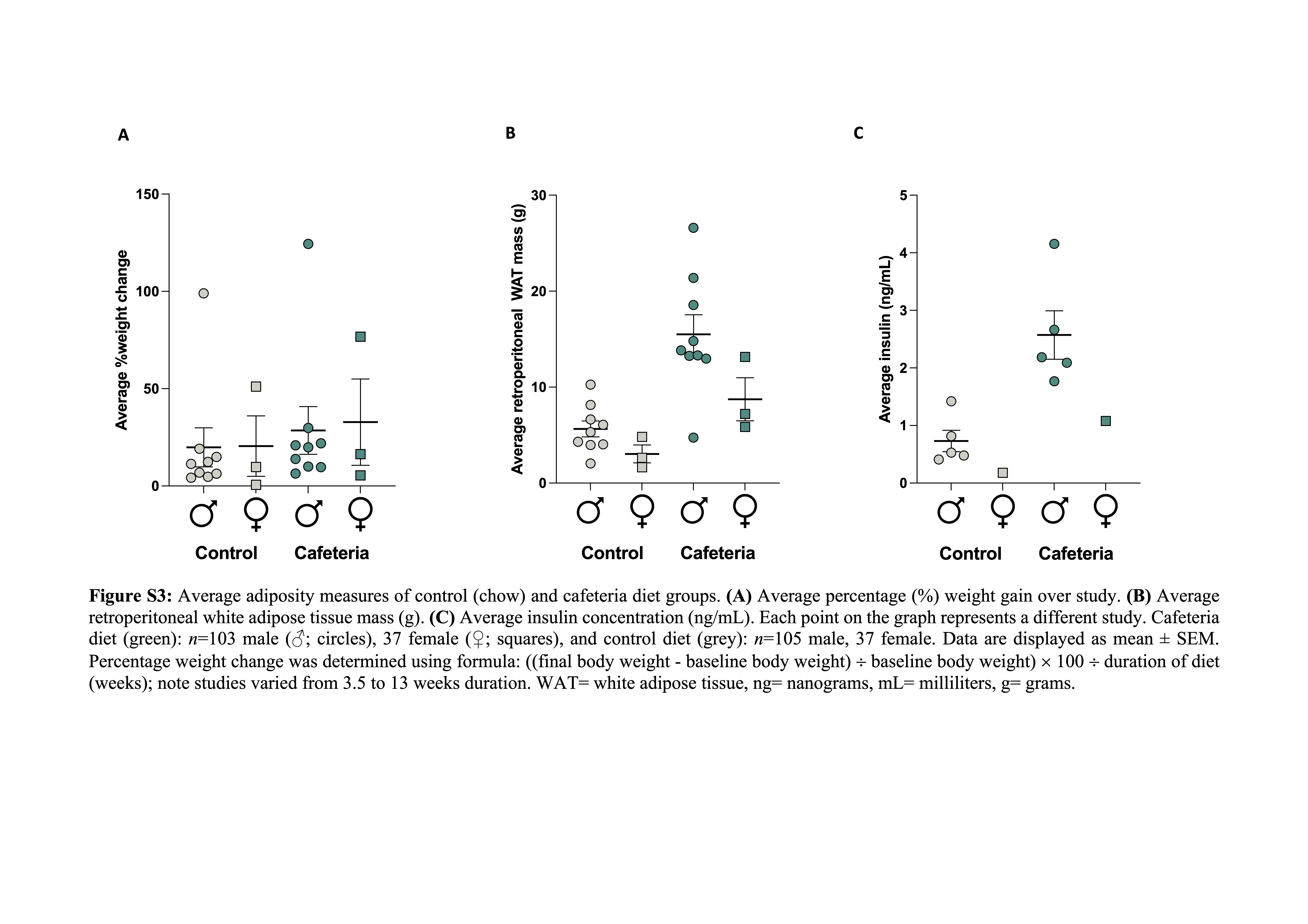

Supplement: Figure S3.jpg [file KGMR_A_2649442_SM2606.jpg]

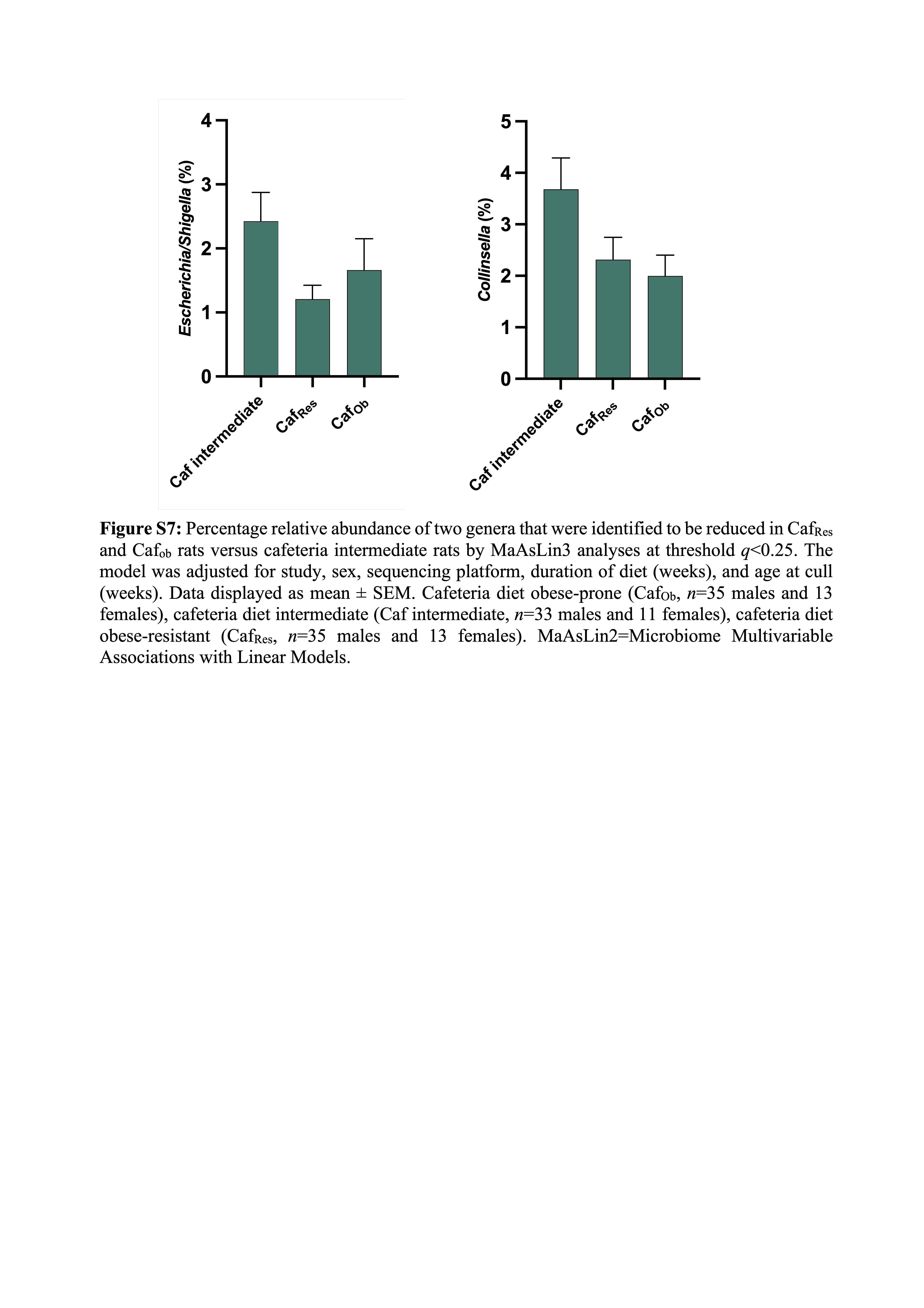

Supplement: Figure S7.jpg [file KGMR_A_2649442_SM2610.jpg]

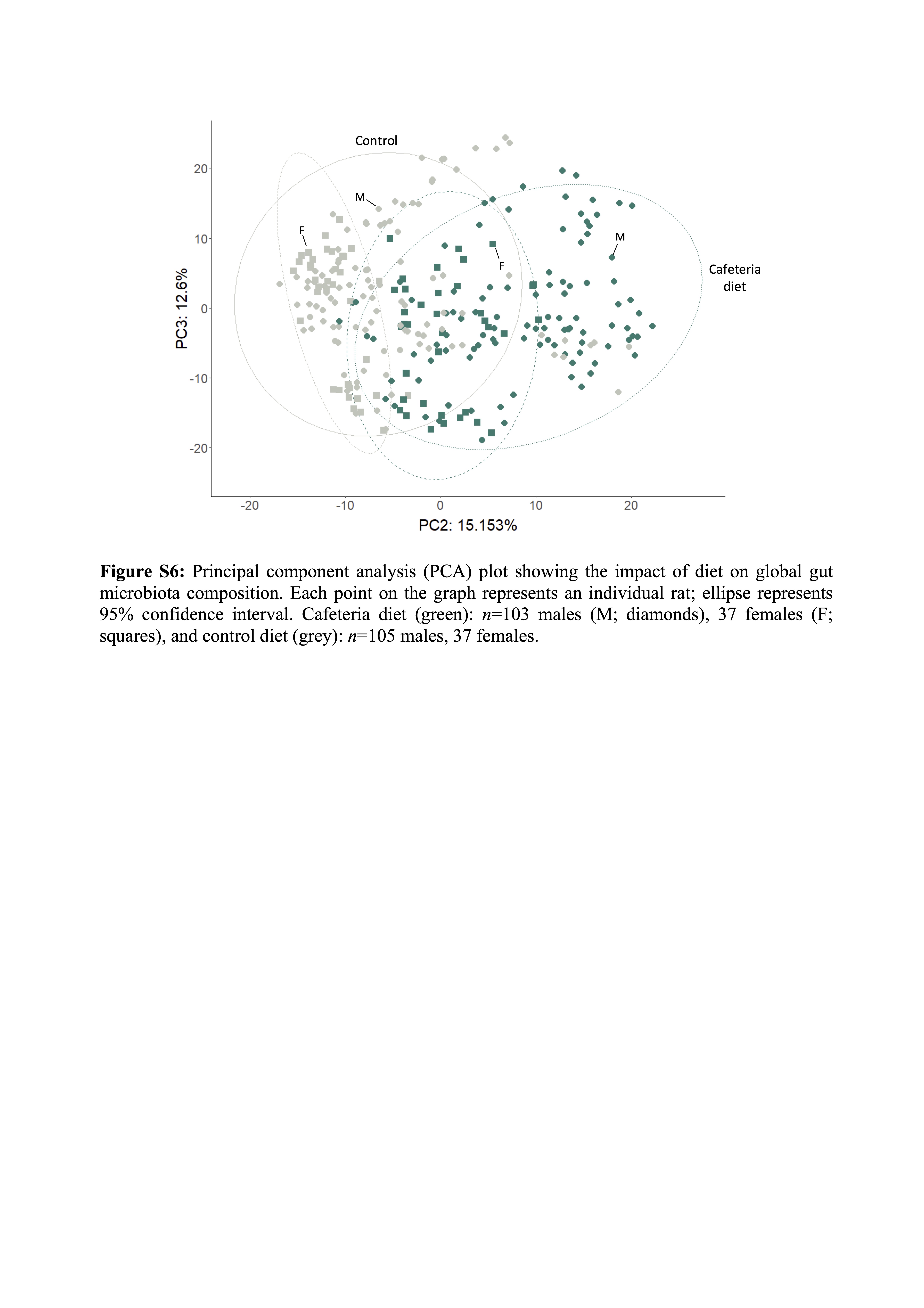

Supplement: Figure S6.jpg [file KGMR_A_2649442_SM2615.jpg]

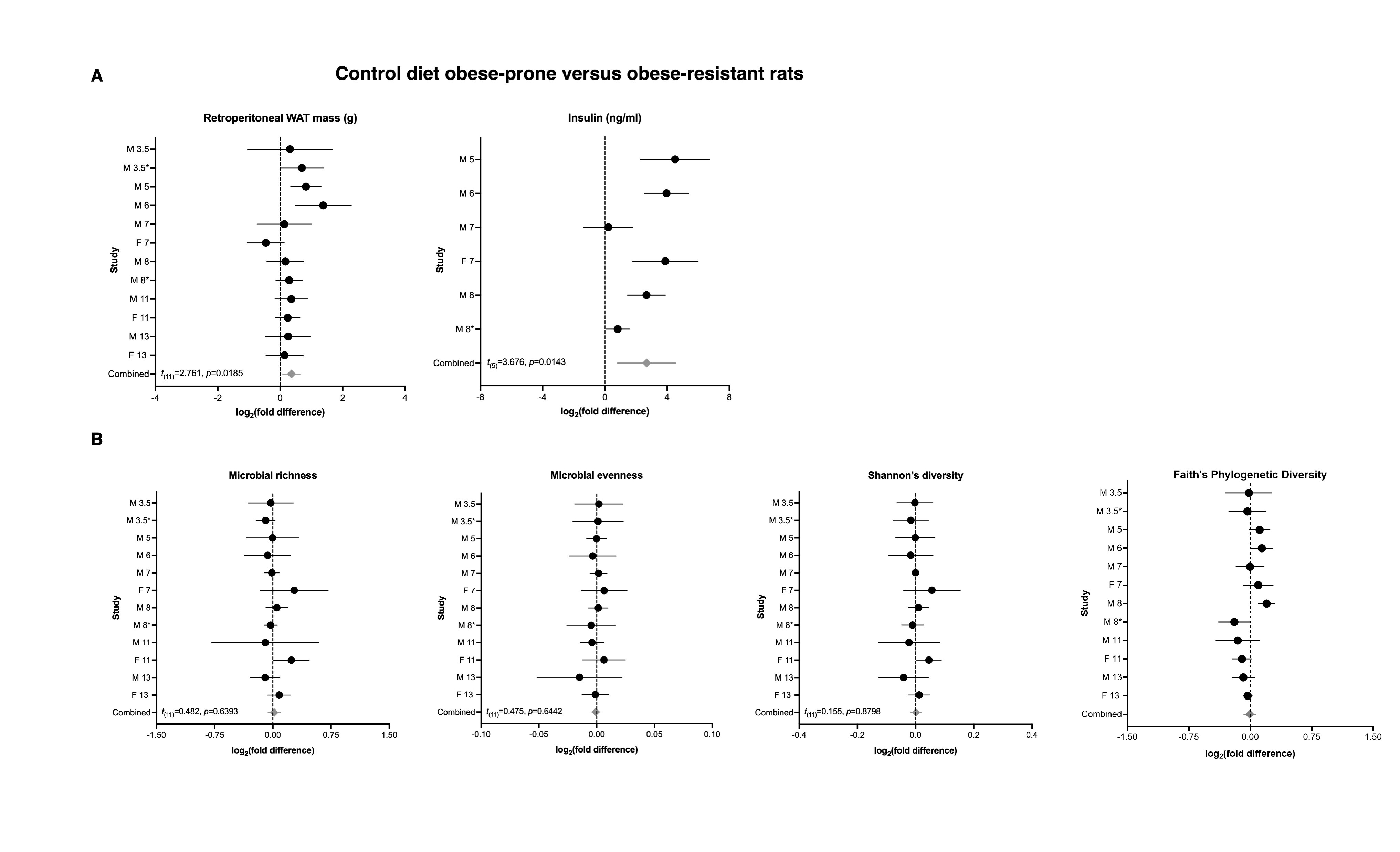

Supplement: Figure S5.jpg [file KGMR_A_2649442_SM2617.jpg]

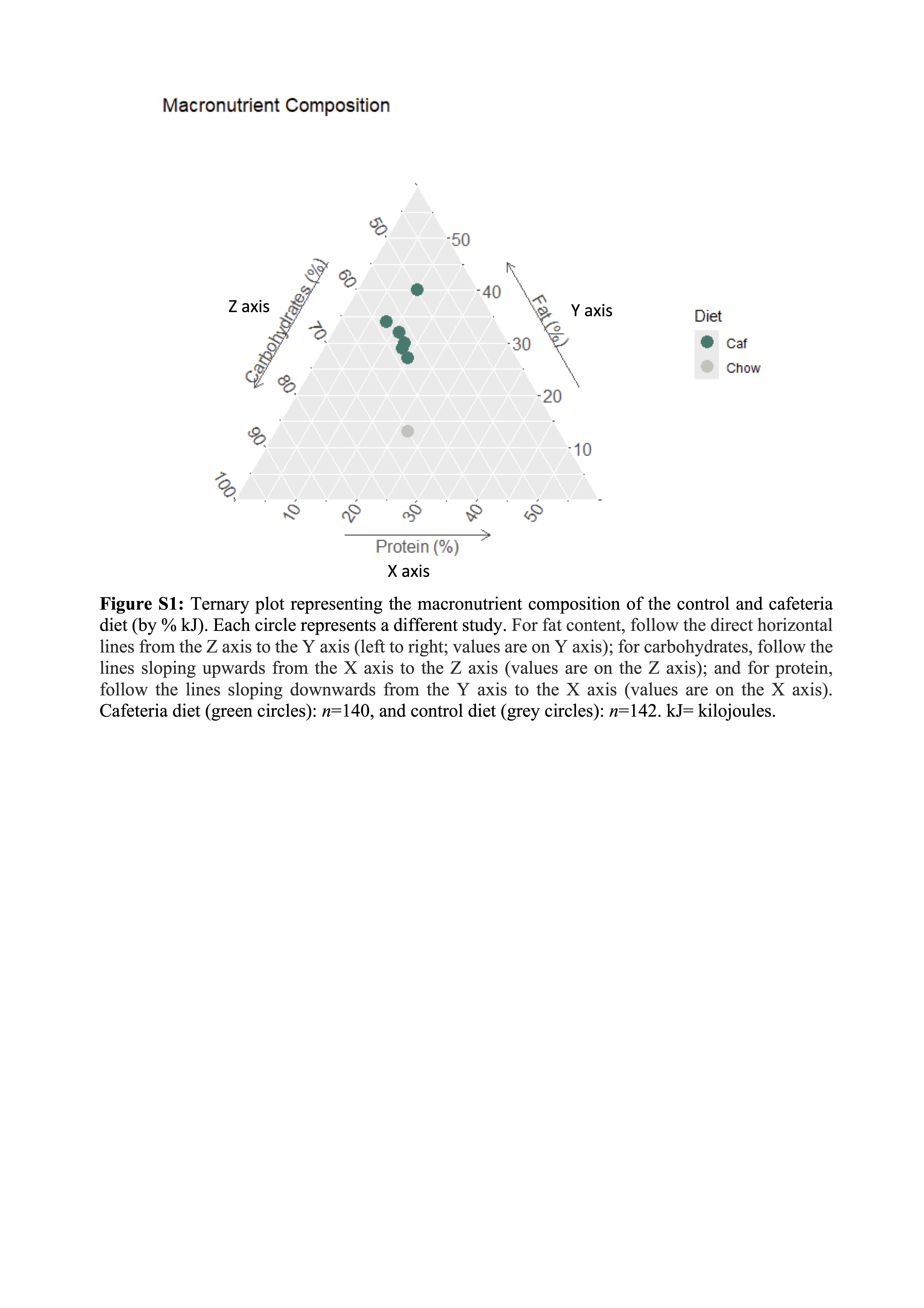

Supplement: Figure S1.jpg [file KGMR_A_2649442_SM2619.jpg]
