# Supplementary material for: Cafeteria diet exposure, and not weight gain propensity, impacts gut microbiota of rats – a within laboratory meta-analysis
Source: Gut Microbes Rep. 2026 Mar 29;3(1):2649442. doi: 10.1080/29933935.2026.2649442 (PMC13037442; doi:10.1080/29933935.2026.2649442)
Supplement: Supplementary Table 1.docx [file KGMR_A_2649442_SM2604.docx]

**Supplementary Table 1:** Summary of body weight measures at baseline and endpoint in cafeteria and control diet rats.

| **Study ID** | **Baseline body weight (g)** | | **Endpoint body weight (g)** | |
| --- | --- | --- | --- | --- |
|  | ***Control*** | ***Caf*** | ***Control*** | ***Caf*** |
| M 3.5 | 377 ± 9.4 | 399.3 ± 4.69 | 440.58 ± 13.75 | 495.7 ± 10.42 |
| M 3.5* | 463.58 ± 15.03 | 473 ± 12.42 | 533.78± 19.66 | 634.07 ± 20.88 |
| M 5 | 373 ± 7.66 | 369.4 ± 7.07 | 502 ± 13.3 | 626.38 ± 15.59 |
| M 6 | 312.76 ± 5.49 | 315.82 ± 4.25 | 526.7 ± 20.1 | 693.99 ± 24.53 |
| M 7 | 202.8 ± 1.71 | 203.75 ± 1.87 | 475.4 ± 14.89 | 625.47 ± 26.17 |
| F 7 | 312.2 ± 12.80 | 306.8 ± 12.07 | 329.9 ± 7.95 | 433.37 ± 17.30 |
| M 8 | 209.43 ± 7.51 | 209.96 ± 7.7 | 412.02 ± 11.71 | 499.1 ± 14.09 |
| M 8* | 204.1 ± 4.62 | 199.7 ± 5.63 | 502.73 ± 8.32 | 625.58 ± 22.4 |
| M 11 | 47.08 ± 1.54 | 47.08 ± 1.54 | 554.91 ± 11.61 | 686.64 ± 22.86 |
| F 11 | 45.37 ± 1.37 | 45.37 ± 1.37 | 298.45 ± 8.85 | 425.36 ± 21.87 |
| M 13 | 314.75 ± 6.05 | 316.83 ± 6.1 | 575.04 ± 14.53 | 728.54 ± 26.53 |
| F 13 | 139 ± 2.7 | 139 ± 2.9 | 316.5 ± 5.7 | 435.1 ± 18.2 |

Data expressed as mean ± SEM. Baseline body weight was measured prior to starting either control (chow) or cafeteria (Caf) diets. Each study is labelled as specified in Table 1 to show sex and diet duration in weeks; for example, M 3.5=male rats fed cafeteria diet for 3.5 weeks. * Indicates a second study of same sex and diet duration. g=grams
