# Supplementary material for: Cafeteria diet exposure, and not weight gain propensity, impacts gut microbiota of rats – a within laboratory meta-analysis
Source: Gut Microbes Rep. 2026 Mar 29;3(1):2649442. doi: 10.1080/29933935.2026.2649442 (PMC13037442; doi:10.1080/29933935.2026.2649442)
Supplement: Supplementary Table 2.docx [file KGMR_A_2649442_SM2607.docx]

**Supplementary Table 2:** Chow and cafeteria diet composition

| **Study ID** | **Energy (kJ)** | | **Protein (%)** | | **Carbohydrate (%)** | | **Fat (%)** | |
| --- | --- | --- | --- | --- | --- | --- | --- | --- |
|  | ***Control*** | ***Caf*** | ***Control*** | ***Caf*** | ***Control*** | ***Caf*** | ***Control*** | ***Caf*** |
| M 3.5 | 361.5 | 914.1 | 22 | 10 | 65 | 50 | 13 | 40 |
| M 3.5* | 417.2 | 1203 | 23 | 9.7 | 65 | 55.8 | 12 | 34.5 |
| M 5 | 376.8 | 1368 | 22 | 10 | 65 | 55 | 13 | 35 |
| M 6 | 343.6 | 1085.3 | 22 | 13 | 65 | 58 | 13 | 29 |
| M 7 | 387.6 | 1004.8 | 22 | 11 | 65 | 57 | 13 | 32 |
| F 7 | 227.2 | 855.5 | 22 | 8 | 65 | 58 | 13 | 34 |
| M 8 | 344.9 | 1216.4 | 22 | 13 | 65 | 57 | 13 | 30 |
| M 8* | 393.1 | 1087 | 23 | 9 | 65 | 58 | 12 | 33 |
| M 11 | 417.2 | 731.8 | 22 | 11.3 | 65 | 58.5 | 13 | 30.2 |
| F 11 | 260.3 | 544.6 | 22 | 11 | 65 | 58.9 | 13 | 30.1 |
| M 13 | 419.5 | 767.1 | 23 | 11 | 65 | 56.6 | 12 | 32.4 |
| F 13 | 257.7 | 775.8 | 22 | 8.7 | 65 | 60.8 | 13 | 30.5 |

Data expressed as average daily intake per rat (energy) or average percentage per rat (macronutrients) per day. Each study is labelled as specified in Table 1 to show sex and diet duration in weeks; for example, M 3.5=male rats fed cafeteria diet for 3.5 weeks. * Indicates a second study of same sex and diet duration. Caf=cafeteria diet, kJ=kilojoules.
