# Supplementary material for: Cafeteria diet exposure, and not weight gain propensity, impacts gut microbiota of rats – a within laboratory meta-analysis
Source: Gut Microbes Rep. 2026 Mar 29;3(1):2649442. doi: 10.1080/29933935.2026.2649442 (PMC13037442; doi:10.1080/29933935.2026.2649442)
Supplement: Supplementary Table 5.docx [file KGMR_A_2649442_SM2608.docx]

**Supplementary Table 5:** Adiposity measures in Caf_Ob_ and Caf_Res_ rats.

| **Study ID** | **%weight change** | | **RP WAT mass (g)** | | **Insulin concentration (ng/ml)** | |
| --- | --- | --- | --- | --- | --- | --- |
|  | ***Caf_Res_*** | ***Caf_Ob_*** | ***Caf_Res_*** | ***Caf_Ob_*** | ***Caf_Res_*** | ***Caf_Ob_*** |
| M 3.5 | 5.41 ± 0.08 | 7.36 ± 0.28 | 3.94 ± 0.17 | 4.52 ± 0.64 | X | X |
| M 3.5* | 8.17 ± 0.42 | 11.17 ± 0.31 | 12.17 ± 1.73 | 15.41 ± 0.97 | X | X |
| M 5 | 12.32 ± 0.42 | 15.31 ± 0.45 | 11.1 ± 2.74 | 15.79 ± 3.10 | 1.19 ± 0.42 | 3.29 ± 0.87 |
| M 6 | 16.98 ± 0.34 | 23.23 ± 1.34 | 17.72 ± 0.65 | 30.05 ± 3.23 | 1.51 ± 0.22 | 8.91 ± 6.08 |
| M 7 | 23.37 ± 1.73 | 37.74 ± 2.04 | 16.68 ± 3.34 | 24.05 ± 2.17 | 1.78 ± 0.46 | 2.68 ± 0.71 |
| F 7 | 3.93 ± 0.66 | 7.1 ± 0.82 | 11.58 ± 0.93 | 15 ± 1.29 | 0.44 ± 0.09 | 1.73 ± 0.39 |
| M 8 | 17.04 ± 0.43 | 25.17 ± 1.48 | 12.45 ± 0.73 | 14.41 ± 1.63 | 2.28 ± 0.69 | 2.75 ± 0.81 |
| M 8* | 17.5 ± 0.94 | 27.8 ± 2.25 | 11.35 ± 1.81 | 20.31 ± 3.14 | 1.17 ± 0.52 | 1.95 ± 0.54 |
| M 11 | 109.52 ± 4.4 | 140.99 ± 6.98 | 10.92 ± 1.51 | 13.48 ± 1.17 | X | X |
| F 11 | 61.61 ± 3.87 | 93.67 ± 1.08 | 5.38 ± 0.67 | 9.52 ± 1.36 | X | X |
| M 13 | 8.76 ± 0.09 | 11.84 ± 0.68 | 19.69 ± 2.76 | 36.64 ± 3.95 | X | X |
| F 13 | 13.3 ± 0.39 | 20.18 ± 0.81 | 4.52 ± 0.20 | 7.63 ± 1.43 | X | X |

Data expressed as mean ± SEM. Retroperitoneal white adipose tissue (RP WAT) mass and insulin concentration were measured at endpoint (at tissue collection). Studies where insulin concentration was not measured are denoted by the symbol “X”. Percentage weight change was calculated from the start of diet to endpoint (cull) and corrected for weeks of diet. Each study is labelled as specified in Table 1 to show sex and diet duration in weeks; for example, M 3.5=male rats fed cafeteria diet for 3.5 weeks. * Indicates a second study of same sex and diet duration. Caf_Ob_=cafeteria diet obese-prone, Caf_Res_=cafeteria diet obese-resistant, ng=nanograms, ml=millilitres, g=grams, %=percentage.
