# Supplementary material for: Cafeteria diet exposure, and not weight gain propensity, impacts gut microbiota of rats – a within laboratory meta-analysis
Source: Gut Microbes Rep. 2026 Mar 29;3(1):2649442. doi: 10.1080/29933935.2026.2649442 (PMC13037442; doi:10.1080/29933935.2026.2649442)
Supplement: Supplementary Table 11.docx [file KGMR_A_2649442_SM2609.docx]

**Supplementary Table 11**: Summary table of all significantly different genera by MaAsLin3 analysis when comparing cafeteria versus control diet rats.

| **Genus** | **Coefficient** | **SE** | **N not 0** | **Individual *p*-value** | **Individual *q*-value** | **Joint *p-*value** | **Joint *q-*value** | **Model** |
| --- | --- | --- | --- | --- | --- | --- | --- | --- |
| *Phascolarctobacterium* | 4.010 | 0.313 | 159 | 2E-18 | 4.61E-16 | 3.74E-18 | 5.85E-16 | Abundance |
| *Clostridiumsensustricto* | -1.367 | 0.320 | 118 | 0.0003 | 0.00169 | 1.90E-16 | 1.98E-14 | Abundance |
| *Odoribacter* | -1.015 | 0.130 | 183 | 6E-07 | 8.09E-06 | 3.29E-16 | 2.58E-14 | Abundance |
| *Blautia* | 2.088 | 0.180 | 253 | 9E-16 | 7.74E-14 | 1.88E-15 | 1.18E-13 | Abundance |
| *Allobaculum* | 1.692 | 0.442 | 106 | 0.0002 | 0.00155 | 3.42E-13 | 1.78E-11 | Abundance |
| *Turicibacter* | -1.812 | 0.344 | 133 | 1E-05 | 0.00011 | 1.30E-12 | 5.82E-11 | Abundance |
| *Bacteroides* | 1.150 | 0.094 | 282 | 2E-12 | 1.17E-10 | 2.13E-12 | 8.33E-11 | Abundance |
| *Anaeroplasma* | -1.819 | 0.257 | 152 | 2E-08 | 4.58E-07 | 8.68E-12 | 3.02E-10 | Abundance |
| *Parabacteroides* | 1.243 | 0.120 | 281 | 9E-12 | 3.93E-10 | 1.76E-11 | 5.49E-10 | Abundance |
| ***Bacteroidota***_unclassified | -1.331 | 0.123 | 213 | 2E-11 | 7.66E-10 | 3.73E-11 | 1.06E-09 | Abundance |
| *Alistipes* | -1.168 | 0.091 | 282 | 4E-11 | 1.53E-09 | 4.35E-11 | 1.13E-09 | Abundance |
| *Anaerostipes* | 1.214 | 0.329 | 74 | 0.0005 | 0.00318 | 8.71E-10 | 1.95E-08 | Abundance |
| *Collinsella* | 0.831 | 0.460 | 94 | 0.0641 | 0.176 | 1.55E-09 | 3.24E-08 | Abundance |
| *Romboutsia* | -1.808 | 0.237 | 228 | 3E-09 | 9.00E-08 | 6.57E-09 | 1.29E-07 | Abundance |
| *Coprobacillus* | 0.258 | 0.812 | 60 | 0.701 | 0.916 | 8.58E-09 | 1.58E-07 | Abundance |
| *Fusicatenibacter* | 1.356 | 0.212 | 140 | 2E-07 | 3.34E-06 | 1.25E-08 | 2.17E-07 | Abundance |
| *Desulfovibrio* | 1.380 | 0.197 | 252 | 1E-08 | 2.68E-07 | 2.28E-08 | 3.76E-07 | Abundance |
| *Parasutterella* | 1.102 | 0.145 | 275 | 1E-08 | 2.90E-07 | 2.59E-08 | 4.05E-07 | Abundance |
| *Peptococcus* | 0.105 | 0.197 | 103 | 0.471 | 0.734 | 8.83E-08 | 1.26E-06 | Abundance |
| *Acetanaerobacterium* | 0.110 | 0.119 | 189 | 0.308 | 0.563 | 1.41E-07 | 1.93E-06 | Abundance |
| *Alloprevotella* | -1.013 | 0.123 | 282 | 2E-07 | 2.61E-06 | 1.59E-07 | 2.07E-06 | Abundance |
| *Bilophila* | 0.639 | 0.224 | 112 | 0.0076 | 0.0329 | 2.18E-07 | 2.53E-06 | Abundance |
| *Subdoligranulum* | 0.464 | 0.237 | 77 | 0.0548 | 0.158 | 2.12E-07 | 2.53E-06 | Abundance |
| *Sutterella* | 1.839 | 0.290 | 167 | 1E-07 | 1.85E-06 | 2.10E-07 | 2.53E-06 | Abundance |
| *Intestinimonas* | -0.856 | 0.090 | 282 | 4E-07 | 5.26E-06 | 3.54E-07 | 3.96E-06 | Abundance |
| *Holdemania* | 0.355 | 0.423 | 53 | 0.357 | 0.617 | 7.25E-07 | 7.57E-06 | Abundance |
| *Rhodospirillaceae*_unclassified | 1.171 | 0.216 | 192 | 3E-06 | 3.90E-05 | 1.24E-06 | 1.25E-05 | Abundance |
| *Mucispirillum* | -0.494 | 0.174 | 194 | 0.0371 | 0.115 | 3.97E-06 | 3.76E-05 | Abundance |
| *Paraprevotella* | 0.676 | 0.209 | 146 | 0.0035 | 0.0168 | 4.19E-06 | 3.76E-05 | Abundance |
| *Staphylococcus* | 1.083 | 0.437 | 48 | 0.0156 | 0.0577 | 4.21E-06 | 3.76E-05 | Abundance |
| *Bacillota*_unclassified | -0.849 | 0.110 | 276 | 3E-06 | 3.51E-05 | 5.85E-06 | 5.08E-05 | Abundance |
| *Butyricicoccus* | 0.598 | 0.121 | 235 | 0.0002 | 0.00126 | 6.53E-06 | 5.53E-05 | Abundance |
| *Lachnospiraceae*_unclassified | -0.712 | 0.084 | 282 | 1E-05 | 0.00012 | 1.25E-05 | 0.0001 | Abundance |
| *Coprococcus* | -1.234 | 0.433 | 53 | 0.0142 | 0.0539 | 1.31E-05 | 0.0001 | Abundance |
| Bacteria_unclassified | -0.862 | 0.128 | 280 | 8E-06 | 8.83E-05 | 1.65E-05 | 0.00013 | Abundance |
| *Butyricimonas* | 0.477 | 0.127 | 243 | 0.0025 | 0.0127 | 1.77E-05 | 0.00013 | Abundance |
| *Murimonas* | 0.638 | 0.371 | 46 | 0.0854 | 0.225 | 3.76E-05 | 0.00027 | Abundance |
| *Clostridiales*_unclassified | -0.646 | 0.077 | 282 | 4E-05 | 0.00037 | 4.31E-05 | 0.0003 | Abundance |
| *Faecalibacterium* | -1.491 | 0.319 | 80 | 0.0001 | 0.00087 | 4.96E-05 | 0.00034 | Abundance |
| *Coriobacteriaceae*_unclassified | -0.674 | 0.122 | 162 | 0.0004 | 0.00227 | 5.45E-05 | 0.00036 | Abundance |
| *Acetatifactor* | -0.906 | 0.160 | 265 | 4E-05 | 0.00037 | 8.58E-05 | 0.00055 | Abundance |
| *Clostridiales Incertae Sedis XIII_*unclassified | 0.285 | 0.174 | 86 | 0.106 | 0.263 | 9.39E-05 | 0.00059 | Abundance |
| *Anaerovorax* | -0.713 | 0.109 | 206 | 7E-05 | 0.00061 | 0.00015 | 0.00092 | Abundance |
| *Sporobacter* | -0.735 | 0.131 | 268 | 0.0002 | 0.00125 | 0.00017 | 0.00102 | Abundance |
| *Sporobacter* | -0.489 | 0.930 | 268 | 0.599 | 0.851 | 0.00017 | 0.00102 | Prevalence |
| *Clostridia*_unclassified | -0.791 | 0.142 | 191 | 0.0001 | 0.00097 | 0.00024 | 0.00142 | Abundance |
| *Lactococcus* | -0.665 | 0.297 | 66 | 0.0625 | 0.173 | 0.00027 | 0.00152 | Abundance |
| *Akkermansia* | -0.054 | 0.271 | 181 | 0.998 | 1 | 0.00032 | 0.0017 | Abundance |
| *Prevotella* | -0.548 | 0.095 | 282 | 0.0013 | 0.00714 | 0.00126 | 0.00589 | Abundance |
| *Enterorhabdus* | -0.371 | 0.168 | 27 | 0.141 | 0.329 | 0.00173 | 0.00797 | Abundance |
| *Morganella* | 0.730 | 0.242 | 41 | 0.0067 | 0.0294 | 0.00289 | 0.01221 | Abundance |
| *Aestuariispira* | 0.519 | 0.155 | 220 | 0.0039 | 0.0186 | 0.00389 | 0.01601 | Abundance |
| *Aestuariispira* | 0.317 | 0.309 | 220 | 0.304 | 0.559 | 0.00389 | 0.01601 | Prevalence |
| *Escherichia/Shigella* | -0.478 | 0.158 | 233 | 0.032 | 0.104 | 0.00396 | 0.0161 | Abundance |
| *Catabacter* | -1.074 | 0.277 | 28 | 0.0025 | 0.0128 | 0.00505 | 0.01999 | Abundance |
| *Bacteroidales*_unclassified | -0.515 | 0.103 | 249 | 0.003 | 0.0146 | 0.0059 | 0.02282 | Abundance |
| *Candidatus Saccharibacteria*_unclassified | -0.544 | 0.400 | 26 | 0.255 | 0.496 | 0.00631 | 0.02379 | Abundance |
| *Ruminococcus* | -0.228 | 0.173 | 207 | 0.397 | 0.656 | 0.00872 | 0.03173 | Abundance |
| *Coprobacter* | 0.295 | 0.241 | 223 | 0.188 | 0.392 | 0.0121 | 0.04063 | Abundance |
| *Deltaproteobacteria*_unclassified | 0.455 | 0.164 | 214 | 0.0134 | 0.0514 | 0.0134 | 0.04403 | Abundance |
| *Deltaproteobacteria*_unclassified | 0.006 | 0.389 | 214 | 0.988 | 1 | 0.0133 | 0.04403 | Prevalence |
| *Christensenella* | 0.247 | 0.238 | 37 | 0.255 | 0.496 | 0.0148 | 0.04831 | Abundance |
| *Peptostreptococcaceae*_unclassified | -0.984 | 0.611 | 17 | 0.161 | 0.358 | 0.0153 | 0.04895 | Abundance |
| *Clostridiaceae*_unclassified | -0.357 | 0.837 | 15 | 0.725 | 0.937 | 0.0170 | 0.05381 | Abundance |
| *Rothia* | 0.194 | 0.152 | 166 | 0.191 | 0.397 | 0.0188 | 0.05829 | Abundance |
| Erysipelotrichaceae*_unclassified* | -0.094 | 0.156 | 184 | 0.835 | 1 | 0.0203 | 0.06178 | Abundance |
| *Hydrogenoanaerobacterium* | -0.383 | 0.274 | 44 | 0.269 | 0.518 | 0.0220 | 0.06628 | Abundance |
| *Clostridium XlVb* | 0.174 | 0.112 | 265 | 0.151 | 0.343 | 0.0237 | 0.06982 | Abundance |
| *Streptococcus* | 0.363 | 0.120 | 200 | 0.0131 | 0.0507 | 0.0259 | 0.0752 | Abundance |

Diet was included as fixed effect, and sequencing depth (minimum reads/sample), study, cage, sex, sequencing platform, duration of diet (weeks), and age at cull (weeks) were included as co-variates in the model. *p*-values were adjusted for multiple comparisons using the Benjamini-Hochberg false discovery rate method, and only differentially abundant genera with a significance value *q*≤0.10 are shown. Coefficients were estimated using both abundance and prevalence models. The individual *q*-value reflects the significance of associations with either differential abundance or prevalence of a given microbial genus, while the joint *q*-value assesses the significance of associations considering both abundance and prevalence. Negative coefficient value denotes decreased abundance and positive coefficient value denotes increased abundance in cafeteria versus control diet rats. SE=standard error. Cafeteria diet: *n*=103 males, 37 females, and control diet: *n*=105 males, 37 females.
