# Supplementary material for: Cafeteria diet exposure, and not weight gain propensity, impacts gut microbiota of rats – a within laboratory meta-analysis
Source: Gut Microbes Rep. 2026 Mar 29;3(1):2649442. doi: 10.1080/29933935.2026.2649442 (PMC13037442; doi:10.1080/29933935.2026.2649442)
Supplement: Supplementary Table 8.docx [file KGMR_A_2649442_SM2611.docx]

**Supplementary Table 8:** Alpha diversity metrics in C_Ob_ and C_Res_ rats.

| **Study ID** | **Microbial richness** | | **Microbial evenness** | | **Shannon’s diversity** | |
| --- | --- | --- | --- | --- | --- | --- |
|  | ***C_Res_*** | ***C_Ob_*** | ***C_Res_*** | ***C_Ob_*** | ***C_Res_*** | ***C_Ob_*** |
| M 3.5 | 292.67 ± 9.83 | 287.37 ± 13.17 | 0.94 ± 0.002 | 0.94 ± 0.0032 | 7.37 ± 0.05 | 7.36 ± 0.07 |
| M 3.5* | 20.99 ± 0.33 | 19.68 ± 0.52 | 0.89 ± 0.0033 | 0.89 ± 0.0042 | 4.33 ± 0.03 | 4.28 ± 0.06 |
| M 5 | 57.94 ± 3.82 | 57.83 ± 4.03 | 0.92 ± 0.0023 | 0.92 ± 0.0017 | 5.56 ± 0.06 | 5.56 ± 0.08 |
| M 6 | 56.39 ± 2.09 | 53.98 ± 3.41 | 0.94 ± 0.0014 | 0.94 ± 0.0041 | 5.57 ± 0.04 | 5.51 ± 0.09 |
| M 7 | 84.11 ± 6.73 | 82.65 ± 1.69 | 0.92 ± 0.0031 | 0.92 ± 0.0015 | 5.93 ± 0.09 | 5.93 ± 0.01 |
| F 7 | 58.94 ± 7.03 | 70.47 ± 6.54 | 0.93 ± 0.0015 | 0.93 ± 0.004 | 5.57 ± 0.14 | 5.79 ± 0.12 |
| M 8 | 61.95 ± 1.65 | 64.16 ± 1.91 | 0.94 ± 0.0006 | 0.94 ± 0.0017 | 5.7 ± 0.03 | 5.74 ± 0.04 |
| M 8* | 66.18 ± 2.68 | 64.73 ± 1.21 | 0.92 ± 0.0019 | 0.92 ± 0.0042 | 5.7 ± 0.04 | 5.66 ± 0.05 |
| M 11 | 167.01 ± 11.47 | 159.94 ± 21.43 | 0.93 ± 0.0024 | 0.93 ± 0.002 | 6.27 ± 0.05 | 6.18 ± 0.14 |
| F 11 | 144.67 ± 8.37 | 170.39 ± 8.57 | 0.93 ± 0.0025 | 0.93 ± 0.0037 | 6.1 ± 0.06 | 6.3 ± 0.06 |
| M 13 | 13 ± 0.51 | 12.13 ± 0.5 | 0.89 ± 0.0049 | 0.88 ± 0.0071 | 3.96 ± 0.02 | 3.85 ± 0.07 |
| F 13 | 75.39 ± 2.14 | 79.86 ± 2.84 | 0.93 ± 0.0013 | 0.93 ± 0.0026 | 5.88 ± 0.03 | 5.93 ± 0.05 |

Data expressed as mean ± SEM. Each study is labelled as specified in Table 1 to show sex and diet duration in weeks; for example, M 3.5=male rats fed cafeteria diet for 3.5 weeks. * Indicates a second study of same sex and diet duration. C_Ob_=control diet obese-prone, C_Res_=control diet obese-resistant.
