# Supplementary material for: Cafeteria diet exposure, and not weight gain propensity, impacts gut microbiota of rats – a within laboratory meta-analysis
Source: Gut Microbes Rep. 2026 Mar 29;3(1):2649442. doi: 10.1080/29933935.2026.2649442 (PMC13037442; doi:10.1080/29933935.2026.2649442)
Supplement: Supplementary Table 4.docx [file KGMR_A_2649442_SM2612.docx]

**Supplementary Table 4:** Alpha diversity metrics in cafeteria and control diet rats.

| Study ID | Microbial richness | | Microbial evenness | | Shannon’s diversity | |
| --- | --- | --- | --- | --- | --- | --- |
|  | ***Control*** | ***Caf*** | ***Control*** | ***Caf*** | ***Control*** | ***Caf*** |
| M 3.5 | 284.77 ± 5.79 | 239.03 ± 12.65 | 0.94 ± 0.0015 | 0.93 ± 0.0036 | 7.34 ± 0.03 | 7.02 ± 0.08 |
| M 3.5* | 20 ± 0.39 | 17.46 ± 0.69 | 0.89 ± 0.0017 | 0.89 ± 0.0030 | 4.28 ± 0.03 | 4.13 ± 0.05 |
| M 5 | 60.35 ± 2.09 | 62.71 ± 2.97 | 0.92 ± 0.0012 | 0.92 ± 0.0026 | 5.61 ± 0.04 | 5.6 ± 0.06 |
| M 6 | 52.83 ± 1.93 | 49.96 ± 1.68 | 0.94 ± 0.0016 | 0.93 ± 0.0023 | 5.49 ± 0.05 | 5.39 ± 0.05 |
| M 7 | 86.4 ± 2.58 | 82.26 ± 2.55 | 0.92 ± 0.0012 | 0.92 ± 0.0026 | 5.97 ± 0.03 | 5.9 ± 0.05 |
| F 7 | 66.51 ± 3.43 | 68.22 ± 2.95 | 0.93 ± 0.0016 | 0.92 ± 0.0035 | 5.71 ± 0.07 | 5.71 ± 0.07 |
| M 8 | 61.43 ± 1.21 | 44.04 ± 1.88 | 0.94 ± 0.0011 | 0.92 ± 0.0028 | 5.68 ± 0.03 | 5.19 ± 0.06 |
| M 8* | 67.47 ± 2.12 | 56.86 ± 4.55 | 0.92 ± 0.0017 | 0.91 ± 0.0039 | 5.7 ± 0.04 | 5.45 ± 0.1 |
| M 11 | 161.08 ± 8.31 | 143.27 ± 6.68 | 0.93 ± 0.0015 | 0.91 ± 0.0039 | 6.21 ± 0.05 | 5.99 ± 0.07 |
| F 11 | 158.32 ± 6.16 | 141.52 ± 5.25 | 0.93 ± 0.0019 | 0.92 ± 0.0032 | 6.19 ± 0.05 | 6 ± 0.05 |
| M 13 | 12.62 ± 0.28 | 14.52 ± 0.21 | 0.89 ± 0.0032 | 0.88 ± 0.0026 | 3.9 ± 0.03 | 4.01 ± 0.02 |
| F 13 | 79.31 ± 1.54 | 73.89 ± 2.40 | 0.93 ± 0.0011 | 0.93 ± 0.0018 | 5.93 ± 0.03 | 5.82 ± 0.04 |

Data expressed as mean ± SEM. Each study is labelled as specified in Table 1 to show sex and diet duration in weeks; for example, M 3.5=male rats fed cafeteria diet for 3.5 weeks. * Indicates a second study of same sex and diet duration. Caf= cafeteria diet.
