# Supplementary material for: Cafeteria diet exposure, and not weight gain propensity, impacts gut microbiota of rats – a within laboratory meta-analysis
Source: Gut Microbes Rep. 2026 Mar 29;3(1):2649442. doi: 10.1080/29933935.2026.2649442 (PMC13037442; doi:10.1080/29933935.2026.2649442)
Supplement: Supplementary Table 6.docx [file KGMR_A_2649442_SM2616.docx]

**Supplementary Table 6:** Alpha diversity metrics in Caf_Ob_ and Caf_Res_ rats.

| **Study ID** | **Microbial richness** | | **Microbial evenness** | | **Shannon’s diversity** | |
| --- | --- | --- | --- | --- | --- | --- |
|  | ***Caf_Res_*** | ***Caf_Ob_*** | ***Caf_Res_*** | ***Caf_Ob_*** | ***Caf_Res_*** | ***Caf_Ob_*** |
| M 3.5 | 232.73 ± 21.47 | 265 ± 19.56 | 0.93 ± 0.0061 | 0.93 ± 0.004 | 6.99 ± 0.14 | 7.19 ± 0.11 |
| M 3.5* | 16.14 ± 0.96 | 18.93 ± 1.1 | 0.88 ± 0.0043 | 0.89 ± 0.0045 | 4.05 ± 0.07 | 4.25 ± 0.08 |
| M 5 | 56.39 ± 3.91 | 63.89 ± 3.83 | 0.91 ± 0.0042 | 0.92 ± 0.0032 | 5.46 ± 0.09 | 5.62 ± 0.08 |
| M 6 | 49.83 ± 2.77 | 51.26 ± 2.92 | 0.93 ± 0.0048 | 0.93 ± 0.0051 | 5.37 ± 0.08 | 5.43 ± 0.09 |
| M 7 | 89.22 ± 2.87 | 77.69 ± 5.98 | 0.93 ± 0.0041 | 0.92 ± 0.0064 | 6.02 ± 0.06 | 5.83 ± 0.12 |
| F 7 | 70.97 ± 2.72 | 62.97 ± 6.73 | 0.93 ± 0.004 | 0.92 ± 0.0081 | 5.79 ± 0.07 | 5.58 ± 0.16 |
| M 8 | 46.96 ± 4.59 | 40.69 ± 1.25 | 0.92 ± 0.0076 | 0.91 ± 0.0018 | 5.28 ± 0.15 | 5.1 ± 0.03 |
| M 8* | 60.25 ± 10.03 | 53.64 ± 9.43 | 0.91 ± 0.0063 | 0.91 ± 0.0095 | 5.5 ± 0.2 | 5.38 ± 0.24 |
| M 11 | 154.51 ± 5.45 | 138.9 ± 16.94 | 0.92 ± 0.0029 | 0.91 ± 0.0079 | 6.13 ± 0.05 | 5.93 ± 0.17 |
| F 11 | 156.67 ± 8.88 | 128.51 ± 2.82 | 0.92 ± 0.0066 | 0.91 ± 0.0034 | 6.12 ± 0.1 | 5.91 ± 0.03 |
| M 13 | 14.79 ± 0.39 | 14.47 ± 0.44 | 0.89 ± 0.004 | 0.88 ± 0.0035 | 4.05 ± 0.01 | 4.01 ± 0.04 |
| F 13 | 75.14 ± 2.89 | 72.72 ± 6.53 | 0.93 ± 0.0028 | 0.92 ± 0.0041 | 5.86 ± 0.05 | 5.77 ± 0.11 |

Data expressed as mean ± SEM. Each study is labelled as specified in Table 1 to show sex and diet duration in weeks; for example, M 3.5=male rats fed cafeteria diet for 3.5 weeks. * Indicates a second study of same sex and diet duration. Caf_Ob_=cafeteria diet obese-prone, Caf_Res_=cafeteria diet obese-resistant.
