# Supplementary material for: Cafeteria diet exposure, and not weight gain propensity, impacts gut microbiota of rats – a within laboratory meta-analysis
Source: Gut Microbes Rep. 2026 Mar 29;3(1):2649442. doi: 10.1080/29933935.2026.2649442 (PMC13037442; doi:10.1080/29933935.2026.2649442)
Supplement: Supplementary Table 10.docx [file KGMR_A_2649442_SM2618.docx]

**Supplementary Table 10**: Rarefaction depths of each study.

| **Study ID** | **Minimum reads/sample** |
| --- | --- |
| M 3.5 | 52617 |
| M 3.5* | 4516 |
| M 5 | 7400 |
| M 6 | 10,038 |
| M 7 | 8346 |
| F 7 | 8346 |
| M 8 | 10,038 |
| M 8* | 83324 |
| M 11 | 30,113 |
| F 11 | 30,113 |
| M 13 | 6430 |
| F 13 | 7649 |

| 16S rRNA sequencing data were processed using MOTHUR. Rarefaction was performed separately within each study, with samples rarefied to the minimum (min) sequencing depth observed in that dataset. The column “Study ID” is formatted as follows for plotting purposes: Each study is labelled to show sex and diet duration in weeks; for example M 3.5=male rats fed cafeteria diet for 3.5 weeks. Where two studies had the same sex and diet duration, an asterisk (*) is used to differentiate the studies. |
| --- |
