# Supplementary material for: Cafeteria diet exposure, and not weight gain propensity, impacts gut microbiota of rats – a within laboratory meta-analysis
Source: Gut Microbes Rep. 2026 Mar 29;3(1):2649442. doi: 10.1080/29933935.2026.2649442 (PMC13037442; doi:10.1080/29933935.2026.2649442)
Supplement: Supplementary Table 9.docx [file KGMR_A_2649442_SM2621.docx]

**Supplementary Table 9**: Beta diversity comparisons at the genus level between groups classified as obese-prone, intermediate, or obese-resistant in each diet group.

|  | **df** | **SS** | **R^2^** | **Pseudo*-F*** | ***p-*value** |
| --- | --- | --- | --- | --- | --- |
| **Main effects** |  |  |  |  |  |
| Diet | 1,276 | 14336.02 | 0.0754 | 22.712 | **0.001** |
| Obesity proneness | 2,276 | 886.89 | 0.0047 | 0.703 | 0.937 |
| Diet: Obesity proneness | 2,276 | 720.02 | 0.0038 | 0.57 | 0.998 |
| **Pairwise comparisons** |  |  |  |  |  |
| C_Ob_ × Control intermediate | 1,92 | 354.25 | 0.0062 | 0.573 | 0.956 |
| C_Ob_ × C_Res_ | 1,94 | 270.64 | 0.0049 | 0.459 | 0.999 |
| C_Ob_ × Caf_Res_ | 1,94 | 5069.51 | 0.0801 | 8.181 | **0.001** |
| C_Ob_ × Caf intermediate | 1,90 | 5377.15 | 0.0866 | 8.538 | **0.001** |
| Caf intermediate × Control intermediate | 1,88 | 5257.29 | 0.0834 | 8.004 | **0.001** |
| Caf_Ob_ × Control intermediate | 1,92 | 4832.04 | 0.0749 | 7.451 | **0.001** |
| Caf_Ob_ × Caf_Res_ | 1,94 | 467.46 | 0.0076 | 0.72 | 0.846 |
| Caf_Ob_ × C_Res_ | 1,94 | 4836.73 | 0.0768 | 7.817 | **0.001** |
| Caf_Ob_ × C_Ob_ | 1,94 | 4833.75 | 0.0763 | 7.76 | **0.001** |
| Caf_Ob_ × Caf intermediate | 1,90 | 561.3 | 0.0093 | 0.849 | 0.625 |
| Caf_Res_ × Control intermediate | 1,92 | 4941.67 | 0.0768 | 7.659 | **0.001** |
| Caf_Res_ × Caf intermediate | 1,90 | 395.62 | 0.0066 | 0.602 | 0.963 |
| C_Res_ × Control intermediate | 1,92 | 362.64 | 0.0064 | 0.591 | 0.962 |
| C_Res_ × Caf intermediate | 1,90 | 5333.45 | 0.0865 | 8.526 | **0.001** |
| C_Res_ × Caf_Res_ | 1,94 | 4972.12 | 0.0791 | 8.077 | **0.001** |

Data were analysed using PERMANOVA (Permutational Multivariate Analysis of Variance) at the genus level, *p*-values were obtained using 1000 permutations, and bold values indicate significance, *p*≤0.05. Caf=cafeteria, Caf_Ob_=cafeteria diet obese-prone, Caf_Res_=cafeteria diet obese-resistant, C_Ob_=control diet obese-prone, C_Res_=control diet obese-resistant, df=degrees of freedom, SS=sum of squares, R^2^=explained variation, Pseudo-F=F value by permutation.
